# ﻿Taxonomic notes on the genus *Baetiella* Uéno, 1931 (Ephemeroptera, Baetidae), with description of three new species from Thailand

**DOI:** 10.3897/zookeys.1200.116787

**Published:** 2024-05-09

**Authors:** Sirikamon Phlai-ngam, Boonsatien Boonsoong, Jean-Luc Gattolliat, Nisarat Tungpairojwong

**Affiliations:** 1 Department of Biology, Faculty of Science, Burapha University, Chonburi 20131, Thailand; 2 Applied Taxonomic Research Center (ATRC), Faculty of Science, Khon Kaen University, Khon Kaen 40002, Thailand; 3 Animal Systematics and Ecology Speciality Research Unit (ASESRU), Department of Zoology, Faculty of Science, Kasetsart University, Bangkok 10900, Thailand; 4 Muséum cantonal des sciences naturelles, département de Zoology, Palais de Rumine, Place Riponne 6, CH-1005 Lausanne, Switzerland; 5 Department of Ecology and Evolution, University of Lausanne, CH-1015 Lausanne, Switzerland; 6 Department of Biology, Faculty of Science, Khon Kaen University, Khon Kaen 40002, Thailand

**Keywords:** COI gene, diversity, mayflies, revision, Southeast Asia

## Abstract

Based on material recently collected in northern Thailand, the present study provides an updated of the genus *Baetiella*, including *Gratia*. It comprises six species in Thailand, three of them being new species: Baetiella (Gratia) narumonae, Baetiella (Gratia) sororculaenadinae, Baetiella (Baetiella) bispinosa, Baetiella (Baetiella) baei**sp. nov.**, Baetiella (Baetiella) lannaensis**sp. nov.** and Baetiella (Baetiella) bibranchia**sp. nov.**Baetiella (Baetiella) baei**sp. nov.** can be distinguished from other species by the reduction of the posteromedian protuberances on abdominal tergites I–III, the asymmetrical coniform terminal segment of labial palp, the distal margin of abdominal sternites VII–X each with a row of long, spatulate setae, the dorsal margin of femur with two long, robust setae distally. Baetiella (Baetiella) lannaensis**sp. nov.** is diagnosed by the posteromedian protuberances present on tergites I–VIII, dorsal margin of femur with a regular row of long, rounded, ciliated setae and body surface covered with numerous, dense, rounded scale-like setae. Baetiella (Baetiella) bibranchia**sp. nov.** can be separated from other species by coxal gills present at the base of forelegs and midlegs. The molecular study based on the mitochondrial gene COI and a larval key to species of Thai *Baetiella* are also provided.

## ﻿Introduction

*Baetiella* is a small genus of Baetidae. It was established by Uéno (1931) for the species *Acentrellajaponica* Imanishi, 1930 (Uéno 1931; Waltz and McCafferty 1987; Shi and Tong 2015a). This genus was subject of several taxonomic revisions: *Baetiella* was considered as a subgenus of Pseudocloeon Klapálek, 1905 then treated as a subgenus of Baetis Leach, 1815 (Kazlauskas 1963; Braasch 1978; Kluge 1983; Tshernova et al. 1986; Tong and Dudgeon 2000; Shi and Tong 2015a).

Waltz and McCafferty (1987) provided morphological evidence in their revision of the status of *Baetiella* that justified recognizing it as a valid and distinct genus, separated from *Pseudocloeon* and *Baetis*, including at that time 12 species across the Palearctic and Oriental regions.

The combination of the following larval characters distinguishes *Baetiella* from other genera of Baetidae: antennae ~1.5× longer than head width; presence of simple submarginal setae on labrum; 2-segmented maxillary palp; labial palp 3-segmented, terminal segment symmetrical or slightly asymmetrical, conical, usually with a small tip at apex, segment II with or without an inner apical lobe; thorax with or without protuberances; femoral villopore present; dorsal margin of tibia with row of dense, fine, simple setae; tarsus without an elongated submarginal seta; tarsal claw with one or two rows of denticles, with a pair of subapical setae; abdominal gills usually present on abdominal segments I–VII; single or paired posteromedian dorsal protuberances present or absent on abdominal tergites; paracercus reduced, shorter than half of the cerci multi-segmented or reduced to one segment; cerci usually longer than body length (Shi and Tong 2015a).

*Baetiella* is occasionally confused with *Gratia* and *Acentrella* Bengtsson, 1912, primarily due to shared larval characters. *Baetiella* can be separated from *Acentrella* by the mouthparts, in particular by the shape of terminal segment of labial palp, which is rounded to truncate in *Acentrella* and usually conical in *Baetiella* (Kluge and Novikova 2011; Shi and Tong 2015a).

Recent revision of the status of *Gratia* seems to indicate that it should be considered as subgenus of *Baetiella*. According to the non-ranking systematics of mayflies, *Baetiella* belongs to the clade Baetiella/g1 within the Baetofemorata; this clade encompasses the three subgenera *Baetiella*, *Gratia*, and *Neobaetiella* (Kluge 2004, 2022b; Kluge and Novikova 2011).At the larval stage, *Gratia* and *Baetiella* are very similar but can be distinguished by differences of setation on the dorsal margin of femur, the submarginal setae on the labrum, the shape of the terminal segment of the labial palp, and the degree of development of the paracercus (Boonsoong et al. 2004; Shi and Tong 2015a, b). To ensure stability and in the absence of a new comprehensive generic revision, we prefer to consider *Gratia* as a subgenus of *Baetiella* for this study.

*Baetiella* currently comprises 19 described species, distributed across the Oriental and Palearctic regions. The latest species to be reported is *Baetiellasubansiri* Vasanth, Selvakumar & Subramanian, 2020, which was discovered in India. *Baetiella* species are distributed across numerous countries, including Japan, Korea, Russia, Mongolia, Tajikistan, Vietnam, China, Nepal, India, and Thailand (Shi and Tong 2015a; Phlai-ngam and Tungpairojwong 2018; Vasanth et al. 2020). Continental China and the Indian subcontinent are the regions with the greatest diversity.

The knowledge of Baetidae in Thailand has advanced significantly in recent decades, both in terms of diversity and systematics. Researchers have discovered numerous new taxa though the intensive, ongoing surveys of mayfly diversity in Southeast Asia. Recently, approximately 17 genera and 26 species of Baetidae were described and recorded in Thailand (Müller-Liebenau and Heard 1979; Thomas 1992; Boonsoong et al. 2004; Kluge and Novikova 2011, 2017; Tungpairojwong and Bae 2015; Phlai-ngam and Tungpairojwong 2018; Suttinun et al. 2018, 2020, 2021, 2022; Kluge 2020; Kluge and Suttinun 2021; Kluge 2022a; Phlai-ngam et al. 2022a, b; Tungpairojwong et al. 2022; Kaltenbach et al. 2023; Kluge et al. 2023). Despite these recent improvements, significant gaps persist, and a large number of taxa remain undescribed. Certain potentially diversified genera, notably *Baetis*, *Labiobaetis* Novikova & Kluge, 1987, and *Nigrobaetis* Novikova & Kluge, 1987, stand out due to their unresolved taxonomic status (Phlai-ngam et al. 2022b).

The genus *Baetiella* has been the subject of limited studies in Thailand and only three species were recorded, including species previously assigned to *Gratia*: Baetiella (Baetiella) bispinosa (Gose, 1980), Baetiella (Gratia) sororculaenadinae (Thomas, 1992), and Baetiella (Gratia) narumonae (Boonsoong & Thomas, 2004). This study provides a comprehensive report on all the species of the genus *Baetiella* (including *Gratia*) distributed in Thailand. Moreover, we offer detailed descriptions and illustrations of three new discovered species. We also provide a diagnostic key to Thai *Baetiella* larvae. Species delimitation and phylogenetic reconstruction were conducted in this study with the support of molecular evidence (mitochondrial COI sequences).

## ﻿Materials and methods

### ﻿Samples

**Figure 1.**
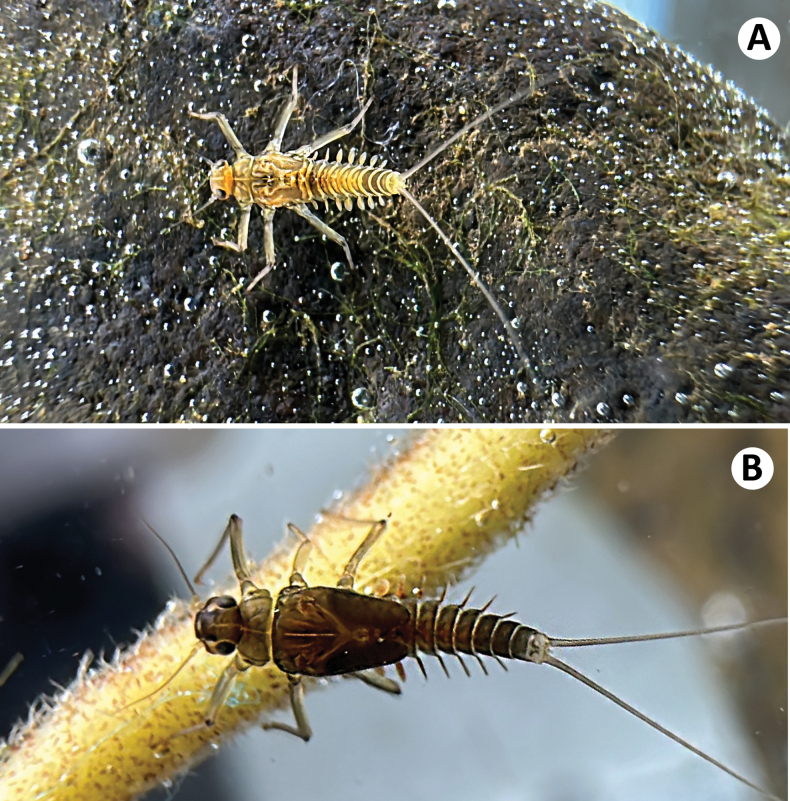
Baetiella (Baetiella) lannaensis sp. nov., female larva **A** early larval stage **B** mature larva.

The larval specimens were handpicked from streams of various orders located in Thailand. The living larvae were photographed with a Canon 700D camera fitted with a 100-mm macro lens, along with an iPhone 14 Pro (Fig. 1). Most of the sampling sites are in the northern region of Thailand (Table 1; Fig. 24). Specimens are housed in the Collection of Aquatic Insects of Department of Biology at Khon Kaen University in Khon Kaen, Thailand (**KKU-AIC**), the collection of the Zoological Museum at Kasetsart University in Bangkok, Thailand (**ZMKU**) and in the Muséum cantonal des sciences naturelles, Department of Zoology, Lausanne, Switzerland (**MZL**). For collecting the mayfly specimens, this research was reviewed and approved by the Institutional Animal Care and Use Committee of Khon Kaen University based on the Ethics of Animal Experimentation of the National Research Council of Thailand (Record No. IACUC-KKU-65/63).

**Table 1.** GPS coordinates of locations of specimens.

Species	Province	GPS coordinates	Altitudes (m a.s.l.)
Baetiella (Baetiella) baei sp. nov.	Chiang Mai	18°32'50.02"N, 98°30'49.79"E	1,359
	Mae Hong Son	19°22'43.79"N, 98°22'32.87"E	855
Baetiella (Baetiella) lannaensis sp. nov.	Chiang Mai	18°32'50.02"N, 98°30'49.79"E	1,359
	Mae Hong Son	19°22'43.79"N, 98°22'32.87"E	855
Baetiella (Baetiella) bibranchia sp. nov.	Chiang Rai	20°00'39.60"N 99°48'14.47"E	476
Baetiella (Baetiella) bispinosa	Chiang Rai	19°31'12.15"N, 99°39'12.59"E	649
	Chiang Mai	19°11'50.51"N, 98°53'13.98"E	362
Baetiella (Gratia) narumonae	Chiang Mai	18°26'22.44"N, 98°35'51.77"E	582
	Chiang Mai	18°29'39.72"N, 98°40'06.65"E	337
	Nan	18°59'47.31"N, 101°12'50.94"E	684
Baetiella (Gratia) sororculaenadinae	Chiang Mai	18°49'02.41"N, 98°55'23.23"E	713
	Mukdahan	16°29'40.48"N, 104°18'47.09"E	208

### ﻿Morphological study

The specimens were preserved in 95% ethanol. Parts of the specimens were dissected and mounted on microscope slides fixed in Euparal or glycerin. Ethanol-preserved specimens were studied using a Nikon SMZ745 stereomicroscope. Drawings of the microscope slides were generated via a camera lucida on an Olympus CH30 compound microscope and subsequently scanned using the Procreate application (iOS application) for illustration. The larvae were captured in photographs using a Nikon Research Stereomicroscope SMZ25 and afterwards processed using NIS-Elements software. The final plates were created and processed using Adobe Photoshop software (http://www.adobe.com). The distribution map was generated with SimpleMappr software (https://simplemappr.net). They were subsequently transferred to absolute ethanol to facilitate the dehydration process to conduct scanning electron microscopy (SEM). The specimens were then dissected, transferred to microtubes, and covered with a fine mesh net (mesh size 60 µm) for drying in a critical point dryer (CPD). The specimens were placed on stubs and coated with a 20-nm gold layer using a Cressington sputter coater. Zeiss LEO 1450 VP was implemented for taking SEM images.

### ﻿Molecular study

The DNA of some specimens was extracted using non-destructive procedures by using PureLink™ Genomic DNA Mini Kit (Invitrogen, Thermo Fisher Scientific, USA), which enabled subsequent morphological examination (more information can be found in the study conducted by Vuataz et al. 2011). A 658 bp fragment of the mitochondrial gene cytochrome oxidase subunit 1 (COI) was amplified using the primers LCO 1490 (GGTCAACAAATCATA­AAGATATTGG) and HCO 2198 (TAAACTTCAGGGTGACCAAAAAATCA) (Folmer et al. 1994). The polymerase chain reaction (PCR) was performed with an initial denaturation temperature of 94 °C for 5 min followed by a total of 35 cycles with a denaturation temperature of 94 °C for 30 sec, an annealing temperature of 48 °C for 40 sec, and an extension at 72 °C for 1 min. The final extension at 72 °C was for 5 min. The PCR products were purified and sequenced by Macrogen, Inc., Korea, and ATGC Co., Ltd., Thailand.

### ﻿Genetic distances and tree analyses

Additional *Baetiella* sequences were obtained from GenBank (http://www.ncbi.nlm.nih.gov/) and Barcode of Life Data System (BOLD) (https://www.boldsystems.org/). The new sequences resulting from this study were also added to the GenBank database (Table 2). All sequences were edited, then aligned using the ClustalW algorithm. Genetic distance among specimens was calculated using MEGA X (Kumar et al. 2018) under the Kimura-2-parameter distances (K2P) model. The CO1 gene trees were obtained using maximum likelihood (ML) and Bayesian inference (BI) approaches.

**Table 2.** Sequenced specimens of *Baetiella* spp. (bold text showing new sequences).

Species	Locality	Source of sequence	Accession number
**Baetiella (Baetiella) baei sp. nov.**	Chiang Mai (Thailand)	GenBank	PP333626, PP333627, PP333628
**Baetiella (Baetiella) lannaensis sp. nov.**	Chiang Mai (Thailand)	GenBank	PP337083, PP337084, PP337085, PP337086
**Baetiella (Baetiella) bibranchia sp. nov.**	Chiang Rai (Thailand)	GenBank	PP341064, PP341065
Baetiella (Baetiella) bispinosa	China	BOLD	ADL1493: XJDQD476-18, XJDQD477-18, XJDQD480-18
Baetiella (Gratia) narumonae	Nan, Chiang Mai, Loei (Thailand)	BOLD, ZMKU	ABU9531: THMAY091-12, THMAY092-12, GN06LE, GN05CR
Baetiella (Gratia) sororculaenadinae	Chiang Mai (Thailand)	ZMKU	GS04CM
*Baetiellatuberculata*	Korea	GenBank	MN442542, MN442543,
			MH823349
*Baetiellajaponica*	Japan	GenBank	KF563015, KF563016
*Baetiella* spp.	India	GenBank	MK393232, MK393233, MK393234
	China	BOLD	ADL1493: XJDQD517-18, XJDQD519-18
	China	BOLD	ADL1407: XJDQD534-18, XJDQD535-18

The ML phylogenetic analysis was conducted using the HKY+GAMMA model as the most appropriate for reconstruction based on the default settings of RAxML-NG on raxmlGUI 2.0 (Edler et al. 2021). Node support values of ML analyses were calculated with 1000 bootstraps (BS) replicates. The BI analysis was performed using MrBayes 3.1.2 (Huelsenbeck and Ronquist 2001). For all data sets, four independent runs of four Monte Carlo Markov chains were conducted for 10,000,000–20,000,000 generations until the average standard deviation of split frequencies decreased to ~0.005, with trees sampled every 1000 generations.

For ML and BI analysis, nodes with BS values ≥ 70% and BI posterior probabilities (PP) ≥ 0.95 were considered highly supported (Hillis and Bull 1993; Felsenstein 2004; Huelsenbeck and Rannala 2004; Mauro and Agorreta 2010). Gene trees were visualized and edited using FigTree v. 1.4 (Rambaut 2014) and Adobe Photoshop software (http://www.adobe.com).

Species delimitation analyses were conducted using two methods (Yaagoubi et al. 2023): the distance-based ASAP approach (Assemble Species by Automatic Partitioning) using the web service available at https://bioinfo.mnhn.fr/abi/public/asap/asapweb.html (Puillandre et al. 2020) and the tree-based mPTP approach (multi-rate Poisson Tree Processes) using the web service available at https://mptp.h-its.org (Kapli et al. 2017).

## ﻿Results

### ﻿Taxonomic account

#### ﻿ Baetiella (Baetiella) baei sp. nov.

Taxon classificationAnimaliaEphemeropteraBaetidae

41299497-B654-5813-A492-EE3DCB35084E

https://zoobank.org/52ED7710-6A18-4404-9C0A-F0C0442EB65F

**Figure 2.**
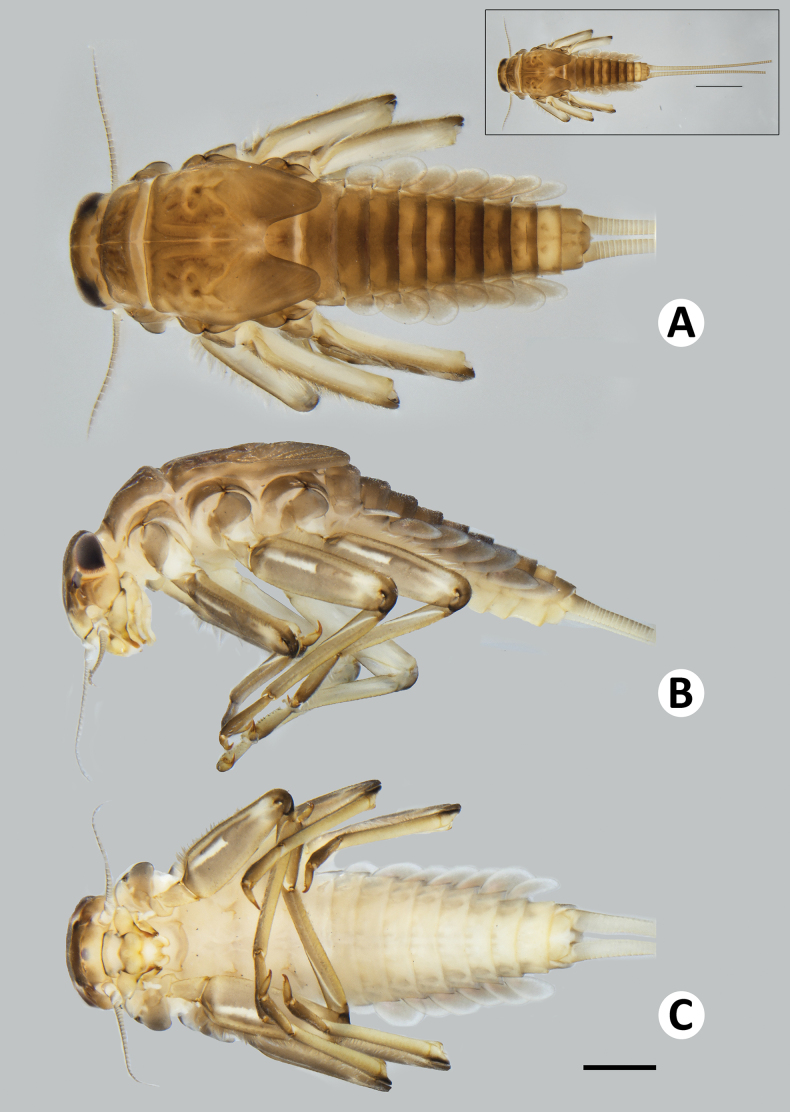
Baetiella (Baetiella) baei sp. nov., larval habitus (paratype) **A** dorsal view **B** lateral view **C** ventral view. Scale bar: 1 mm.

**Figure 3.**
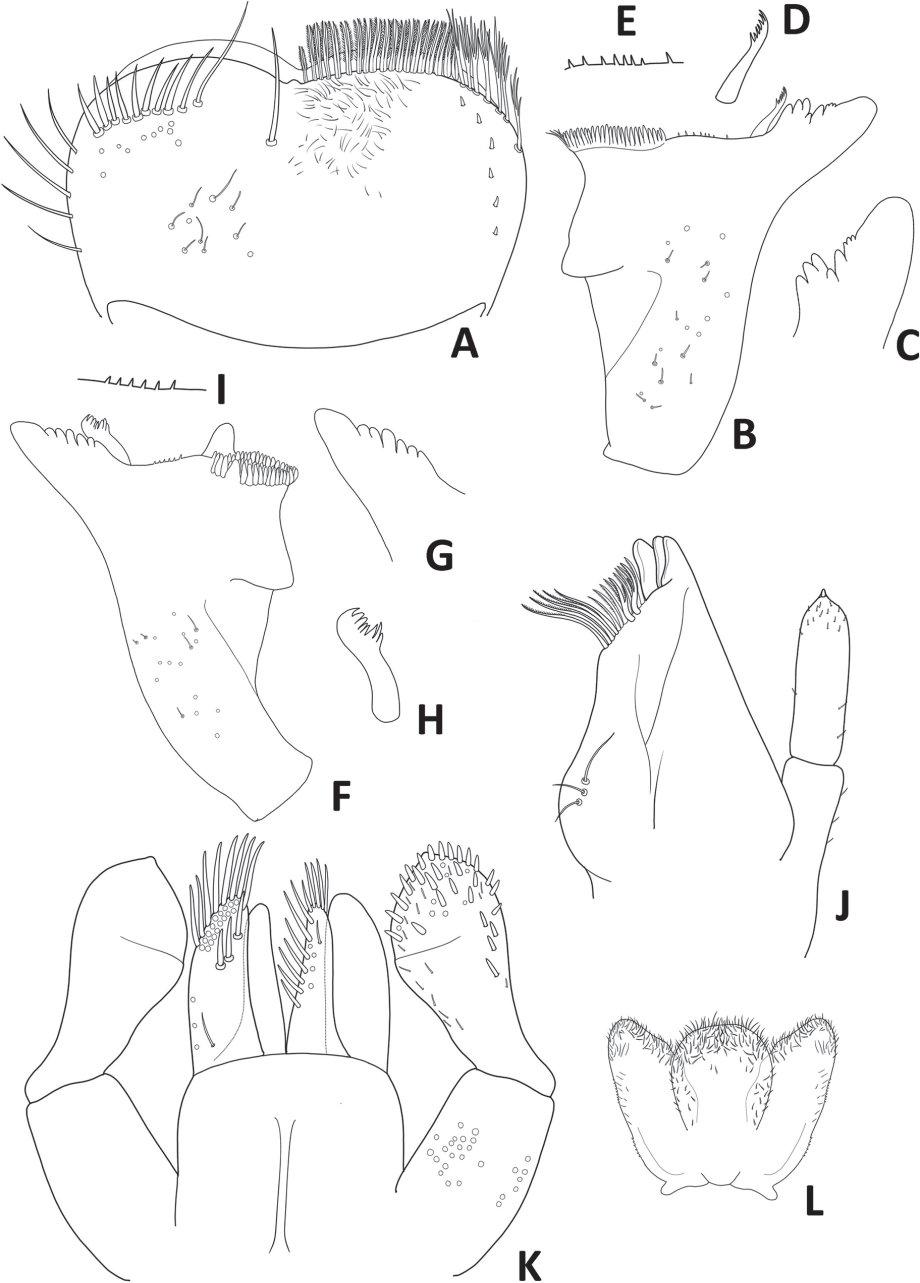
Baetiella (Baetiella) baei sp. nov., larval morphology **A** labrum **B** right mandible **C** right incisors **D** right prostheca **E** margin of right mandible **F** left mandible **G** left incisors **H** left prostheca **I** margin of left mandible **J** maxilla **K** labium **L** hypopharynx.

**Figure 4.**
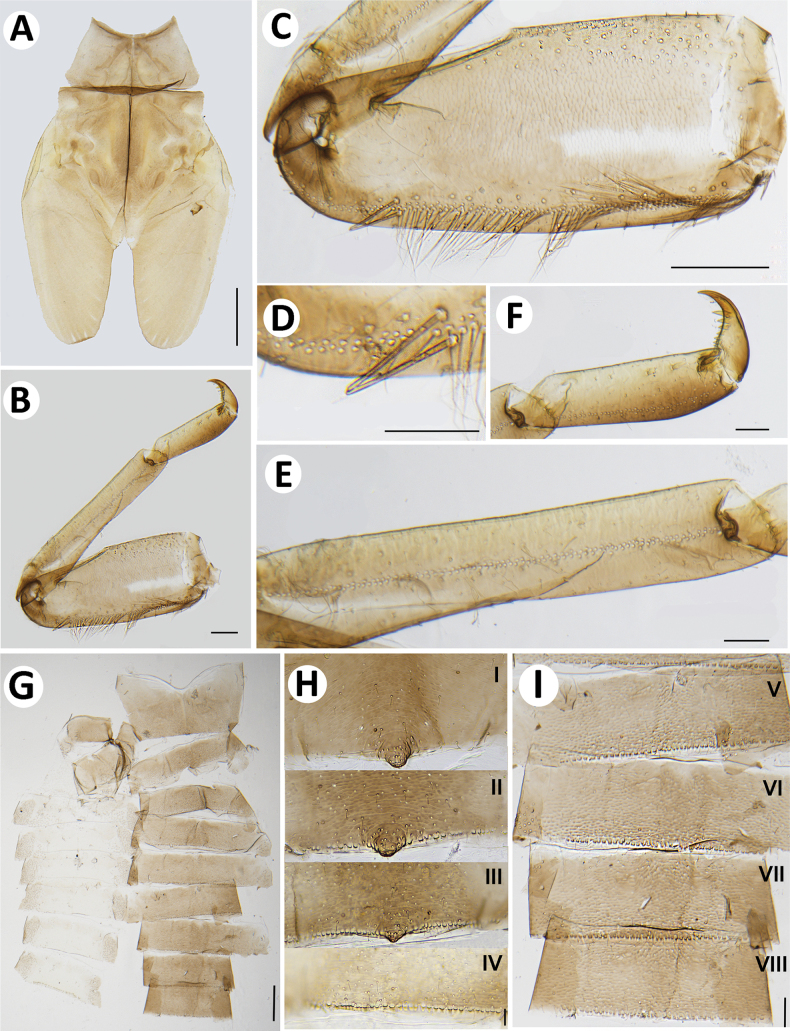
Baetiella (Baetiella) baei sp. nov., larval morphology **A** pronotum and metanotum **B** foreleg **C** femur **D** setae on distal margin of femur **E** tibia **F** tarsus and claw **G** abdomen **H** tergites I–IV **I** tergites V–VIII. Scale bars: 0.5 mm (**A**); 0.1 mm (**B, C, G**); 0.05 mm (**D, E, F, H, I**).

**Figure 5.**
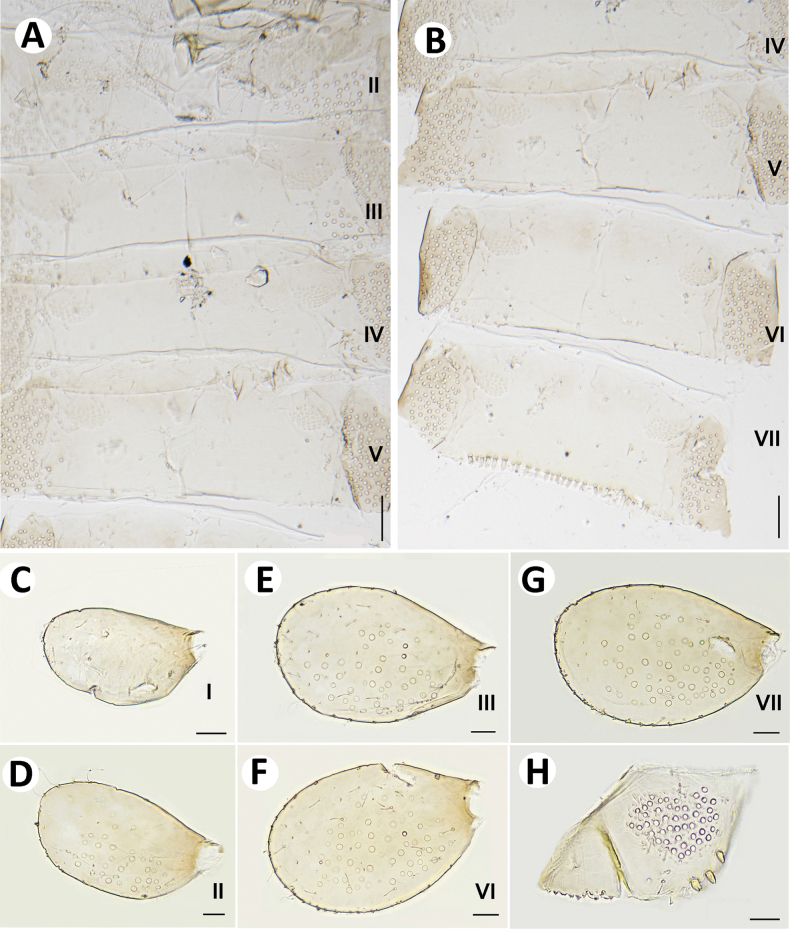
Baetiella (Baetiella) baei sp. nov., larval morphology **A** sternites II–V **B** sternites VI–VIII **C** gill I **D** gill II **E** gill III **F** gill VI **G** gill VII **H** paraproct. Scale bars: 0.05 mm (**A, B**); 0.02 mm (**C–H**).

**Figure 6.**
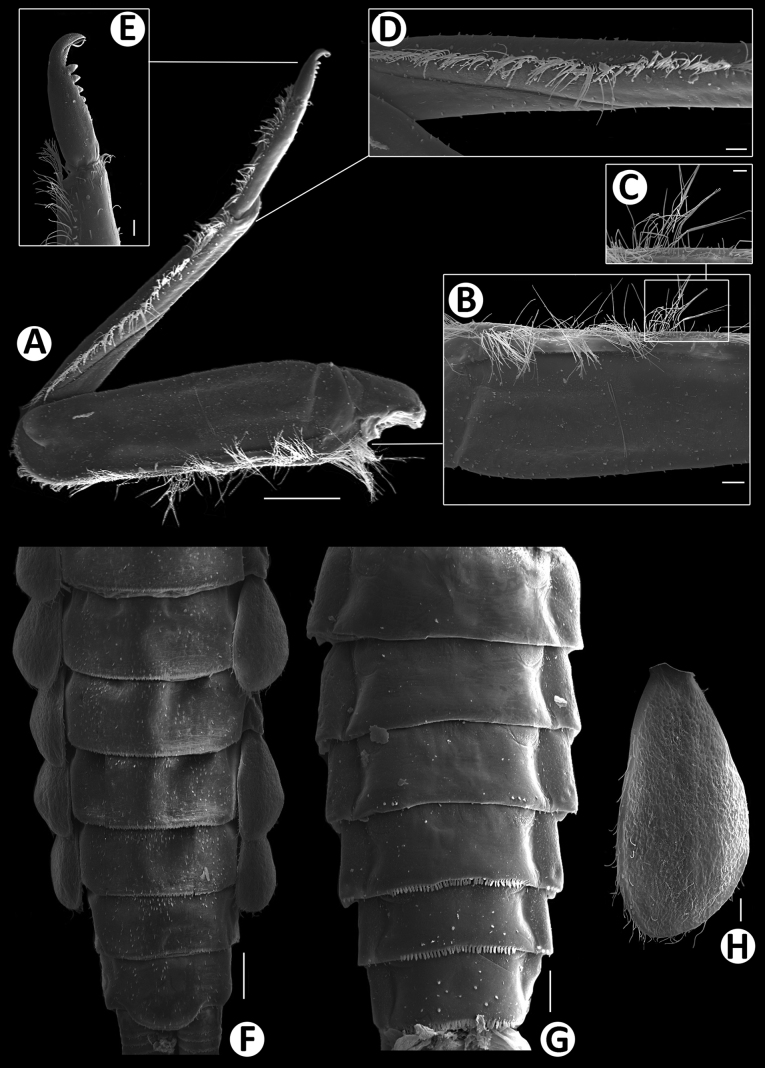
Baetiella (Baetiella) baei sp. nov., larval morphology (SEM) **A** foreleg **B** femur **C** setae on dorsal margin of femur **D** tibia **E** distal tarsus and claw **F** tergites **G** sternites **H** gill IV. Scale bars: 200 µm (**A**); 30 µm (**B, D**); 20 µm (**C, E, H**); 100 µm (**F, H**).

**Figure 7.**
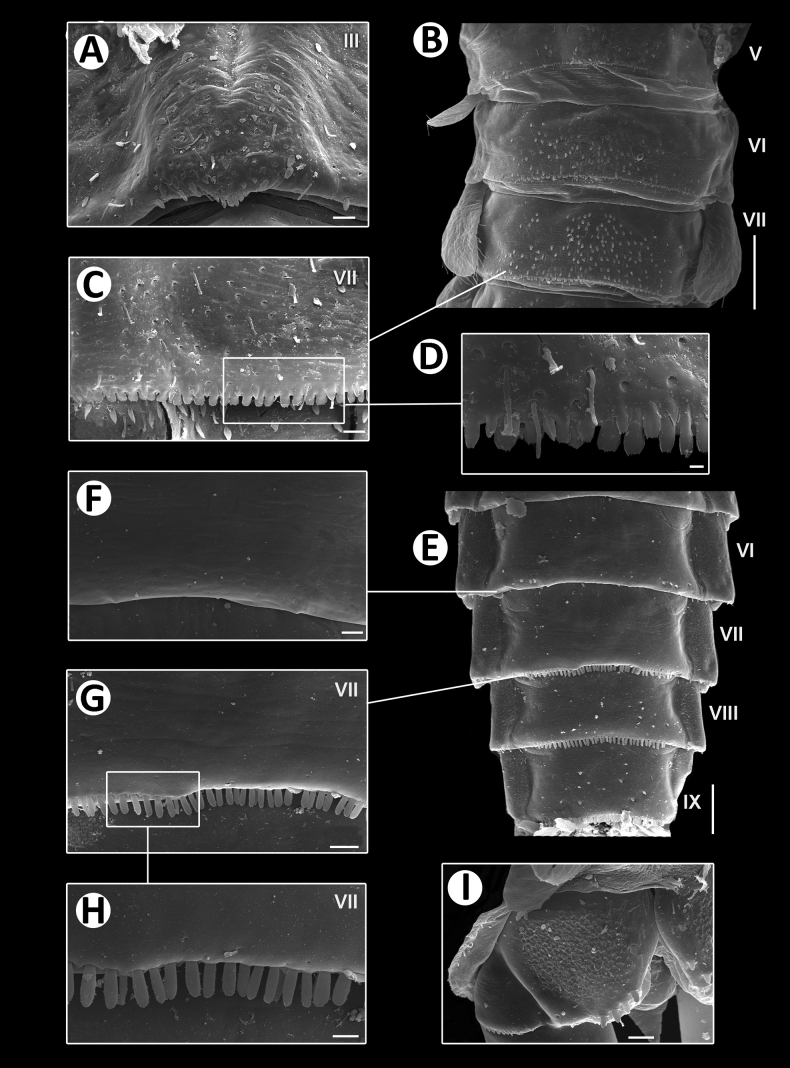
Baetiella (Baetiella) baei sp. nov., larval morphology (SEM) **A** tergite III **B** tergites V–VII **C** tergite VII **D** posterior margin of tergite VII **E** sternites VI–IX **F** sternite VI **G** sternite VII **H** posterior margin of sternites VII **I** paraproct. Scale bars: 10 µm (**A, C, H**); 100 µm (**B**); 3 µm (**D**); 100 (**E**); 20 µm (**F, G, I**).

Figs 2 , 3 , 4 , 5 , 6 , 7

##### Type material examined.

***Holotype*.** Thailand, One larva on slide (KKU-AIC), Chiang Mai, Chom Thong district, Ban Luang, Siribhum waterfall, 18°32'50.02"N, 98°30'49.79"E, 1,359 m, 27.XII.2022, S. Phlai-ngam and B. Boonsoong leg. ***Paratypes*.** One larva on slide, 2 larvae on stubs, 3 larvae in alcohol, same data as holotype (KKU-AIC).

##### Other material examined.

Five larvae in alcohol (KKU-AIC), Thailand, Mae Hong Son, Pai district, Mo Pang waterfall, 19°22'43.79"N, 98°22'32.87"E, 855 m, 10.V.2023., S. Phlai-ngam leg. Six larvae in alcohol (two in MZL: GBIFCH01118450; four in ZMKU), Chiang Mai, Chom Thong district, Ban Luang, Siribhum waterfall, 18°32'50.02"N, 98°30'49.79"E, 1,359 m, 17.XII.2020, B. Boonsoong leg.

##### Diagnosis.

The larvae of *Baetiellabaei* sp. nov. are similar to *Baetiellamarginata* Braasch, 1983 and *Baetiellamuchei* Braasch, 1978; they share the reduction of their posteromedian protuberances of distal tergites (Braasch 1978, 1983). In addition, *Baetiellabaei* sp. nov. can be distinguished from other species by the combination of characters (i) distal margin of tergites I–III with a small, reduced, single posteromedian protuberance; (ii) terminal segment of labial palp rounded, asymmetrical, almost fused with segment II and small tip at apex; (iii) labial palp segment II with very small inner apical lobe; (iv) edge between mola and prostheca of both right and left mandibles with a row of small spines; (v) hindwing pad reduced ~2.5–3.0× longer than width; (vi) dorsal margin of femur with a pair of long, stout, simple, subapical setae distally; (vii) distal margin of tergite II–X with multi-dentated, blunt denticles; (viii) distal margin of sternites I–VI smooth without denticles while sternites VII–X with a row of long, spatulate, blunt denticles; (ix) inner margin of paraproct smooth with 3–5 oval scale-like setae along margin; (x) paracercus reduced to one segment.

##### Description.

Coloration (Fig. 2A). Head dorsally brownish, with darker brown mark at frontal suture. Thorax dorsally brownish with darker brown pattern. Legs brownish; dorsal surface brownish, pale brown ventrally; dorsal surface of femur with pale brown longitudinal stripe along dorsal margin, tarsus and claw distally darker brown (Fig. 2B). Abdominal tergites brownish with dark brown pattern; sternites pale brown (Fig. 2C). Caudal filaments brownish.

***Body*.** Dorsoventrally somewhat flattened (Fig. 2B). Paracercus reduced to one segment, cerci subequal to body length .

***Head*** ~2× wider than long.

Antenna (Fig. 2A). Length ~2× of head length; scape, pedicel and flagellum without process, without scale bases and spines, covered with scattered, fine setae; flagellum covered with scattered, fine setae in each segment.

***Mouthparts*.** Labrum (Fig. 3A). Broad, slightly rectangular; ~2× wider than long; each half of dorsal surface with one central seta and a row of ten long, simple, robust, submarginal setae, proximal part with scattered, fine, simple setae; distal margin with anteromedian notch shallow, lateral margin with a row of long, fine, simple, setae; ventral surface with a row of feathered, setae along distal margin, distolateral margin with a row of feathered, setae and a row of six, short, simple, robust setae near lateral margin, distal part with patch of dense, fine, hair-like setae.

Right mandible (Fig. 3B–E). Outer and inner incisors partially separated with visible line, incisors well developed (Fig. 3B), outer incisor with three denticles and inner incisor with four denticles (Fig. 3C); right prostheca (Fig. 3D) slender with denticles apically; edge between mola and prostheca smooth with a row of eight small spines (Fig. 3E); apex of mola with tuft of spines-like setae; proximal surface with scattered, short, fine, simple setae.

Left mandible (Fig. 3F–I). Outer and inner incisors almost completely fused (Fig. 3F), with seven denticles apically (Fig. 3G), prostheca robust, apically with small denticles and comb-shaped structure (Fig. 3H); edge between mola and prostheca smooth with a row of six small spines (Fig. 3I); proximal surface with scattered, short, fine, simple setae.

Maxilla (Fig. 3J). Galea lacinia with three blunt, robust canines and a canine-like dentiseta; inner dorsal row of setae with two bifid pectinate dentisetae; inner ventral row of robust, simple pectinate setae medially, with long robust, simple pectinate setae distally; with a row of three long setae on basis of galea lacinia. Maxillary palp 2-segmented, relative short, not reaching tip of galea lacinia, surface with scattered small, hair-like setae; outer margin of segment II with distinct concavity; distal segment with distinct, small tip at apex and small, hair-like setae.

Labium (Fig. 3K). Glossa basally broad, narrower toward apex, glossa subequal in length to paraglossa, inner margin with a row of eight stout, simple setae, apical margin with three or four long, robust, blunt, simple setae, with a long, fine, pointed seta subapically; paraglossa sub-rectangular, broader than glossa, apical margin with three rows of long, stout, simple setae, inner margin with a row of medium, stout, simple setae, dorsal surface with three medium, stout, simple setae subapically and proximal part with long, fine, pointed, simple setae. Labial palp 3-segmented; terminal segment rounded, asymmetrical; almost fused with segment II, with small tip at apex; segment II with very small inner apical lobe, a row of four medium, acute, robust setae along outer margin; ventral surface covered with scattered short, robust, simple setae and fine setae; segments I and III with micropores.

Hypopharynx (Fig. 3L). Lingua subequal to superlingua, apically rounded, with apical tuft of long, fine, simple setae; superlingua with distal margin slight­ly truncate, covered with fine, simple setae.

***Thorax*** (Fig. 4A).

Pronotum and mesonotum with small, reduced tubercles.

Hindwing pad reduced, ~2.5–3.0× longer than width.

Legs (Figs 4B–F, 6A–E). Foreleg. Femur (Figs 4C, 6B) length ~2.5× maximum width. Dorsal margin with row of dense, long, fine, simple setae, length ~1/3 to 1/2 of femur width, decreasing at distal part (Fig. 6B, C), with a pair of long, stout, simple, subapical setae distally (Fig. 4C, D). All dorsal and ventral margins with a scattered row of short, robust setae. Femoral villopore present. Dorsal surface with scattered fine setae.

Tibia (Figs 4E, 6D) with a row of long, fine, simple setae dorsally, dorsal and ventral margins with a scattered row of short, robust setae.

Tarsus (Figs 4F, 6E) with a row of long, fine, simple setae dorsally, with a row of four robust, blunt setae ventrally increasing in size on distal part. Tarsal claw with a row of seven denticles, with pair of subapical setae. All legs without coxal gill.

Midlegs and hindlegs as forelegs.

***Abdomen*** (Fig. 4G). Distal margin of abdominal tergites I–III with a single, reduced, posteromedian protuberance. Distal margin of tergites IV–X without posteromedian protuberance.

Abdominal tergites I–X (Figs 4G–I, 6F, 7A–D): distal margin with multi-dentated, blunt spines, surface with scattered, fine setae.

Abdominal sternites (Figs 5A, B, 6G, 7E–H). Distal margin of sternites I–VI smooth without denticles or scale-like setae (Fig. 7E, F); distal margin of sternites VII–X with a row of long, spatulate, blunt denticles (Fig. 7E, G, H).

Gills (Figs 5C–F, 6H). Seven pairs of gills present on abdominal tergites I–VII, oval and without visible tracheation; gill I smaller than other gills (Fig. 5C), surface with scattered, fine setae and a few micropores; gills II–VII with numerous, fine setae and several micropores on surface; gill margin smooth with hair-like setae and scattered small spines (Figs 5C–F, 6H); coloration reddish to brown medially, translucent on outer margin.

Paraproct (Figs 5H, 7I). Margin smooth with three to five scale-like setae, surface with numerous micropores and scattered fine setae; margin of cercotractor with 12–14 spines. Median caudal filament reduced to one segment, cerci subequal in length with body length.

**Imago.** Unknown.

##### Ecological notes.

The larvae of *Baetiellabaei* sp. nov. were collected in Siribhum and Mo Pang waterfalls close to the headwater located in Chiang Mai and Mae Hong Son Provinces (northern Thailand). These sampling sites were situated at medium to high altitudes (870–1,360 m a.s.l.). The substrate types were dominated by boulders, cobbles, pebbles, gravel, and a sand bottom. The larvae were found on the surface boulders in medium to fast-flowing water, ~0.5 m/s–0.7 m/s (Fig. 23).

##### Etymology.

This specific epithet, *baei* (masculine), is dedicated to Professor Dr. Yeon Jae Bae (Division of Environmental Science and Ecological Engineering, Korea University, South Korea) in honor of his outstanding accomplishments as the pioneer researcher and influential leader of aquatic insect and benthological studies of Asia.

##### Distribution.

Chiang Mai and Mae Hong Son Provinces (northern Thailand).

##### Remarks.

The differences between this newly discovered species and *Baetiellamarginata* is readily apparent in the absence of posteromedian protuberances on the distal margin of all tergites of *B.marginata*. The paracercus of Baetiella (Baetiella) baei sp. nov. is reduced to a single cone-shaped segment, whereas in *B.marginata*, it reduces to ~15 segments. Furthermore, Baetiella (Baetiella) baei sp. nov. has a row of spines on the edge between prostheca and mola of both mandibles, while the edges of *B.marginata* are devoid of any spines or serration and appear smooth (Braasch 1983; Shi and Tong 2015a; Vasanth et al. 2020).

*Baetiellamuchei* also presents important similarity to Baetiella (Baetiella) baei sp. nov., mainly characterized by the reduction of the paracercus to one segment. The diagnostic characters separating these species are: (i) the distal margin of tergites of *B.muchei* lacks posteromedian protuberances, comparable to *B.marginata*; (ii) the terminal segment of the labial palp in *B.muchei* is conical in shape, with an apical tip at the apex, while our new species presents an asymmetrical shape; (iii) the setation of the dorsal margin of the femur differs between Baetiella (Baetiella) baei sp. nov. and *B.muchei*, as the former possesses a pair of long, stout, simple, subapical setae distally; (iv) the distal margin of sternites VIII–X in Baetiella (Baetiella) baei sp. nov. with a row of long, spatulate, blunt denticles; (v) the inner margin of the paraproct in this new species is smooth with 3–5 distinct scale-like setae along the margin, these scale-like setae are lacking in *B.muchei*. Additionally, the gills of *B.muchei* reveal a longitudinal brown band in the middle area, while gills of Baetiella (Baetiella) baei sp. nov. display a rounded and oval brown shading in the same location (Fig. 2A). Moreover, the edge between prostheca and mola of mandible of *B.muchei* are also smooth without any spines like *B.marginata* that contrasts with the new species. (Braasch 1978; Shi and Tong 2015a; Sivaruban et al. 2024).

#### ﻿ Baetiella (Baetiella) lannaensis sp. nov.

Taxon classificationAnimaliaEphemeropteraBaetidae

F4148072-D4EF-5554-BCF2-9FBFE9F94B50

https://zoobank.org/97A175BF-6310-4202-8A90-95680662C97D

**Figure 8.**
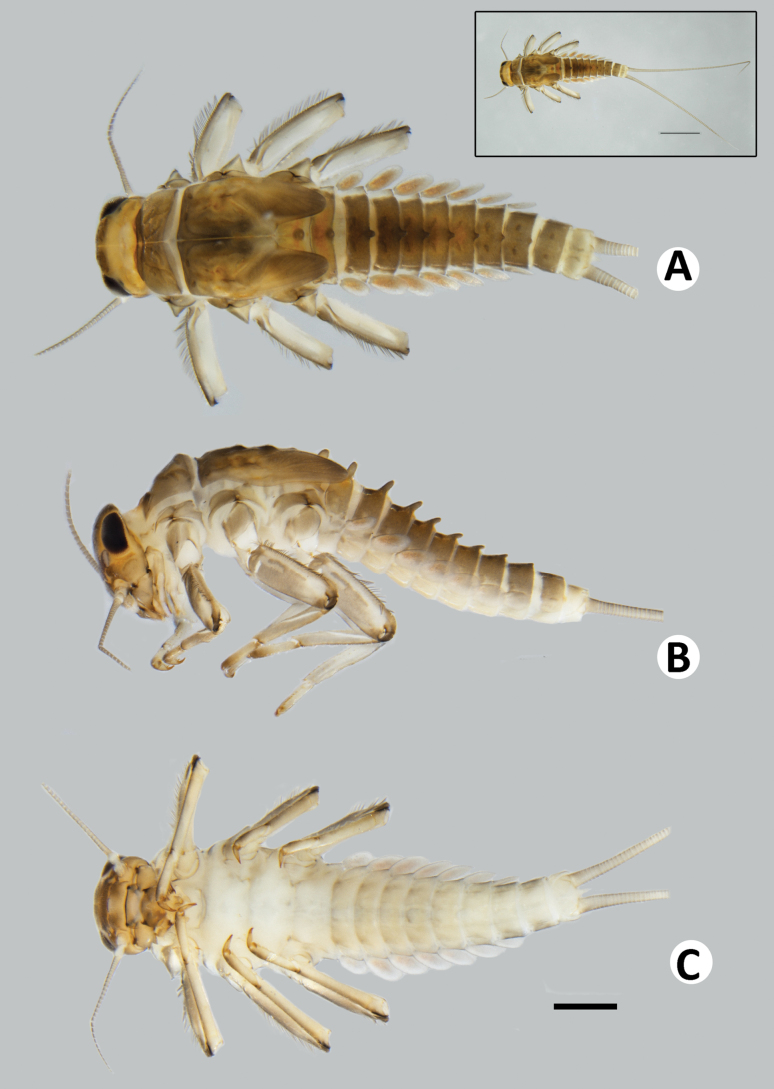
Baetiella (Baetiella) lannaensis sp. nov., larval habitus (paratype): **A** dorsal view **B** lateral view **C** ventral view. Scale bar: 1 mm.

**Figure 9.**
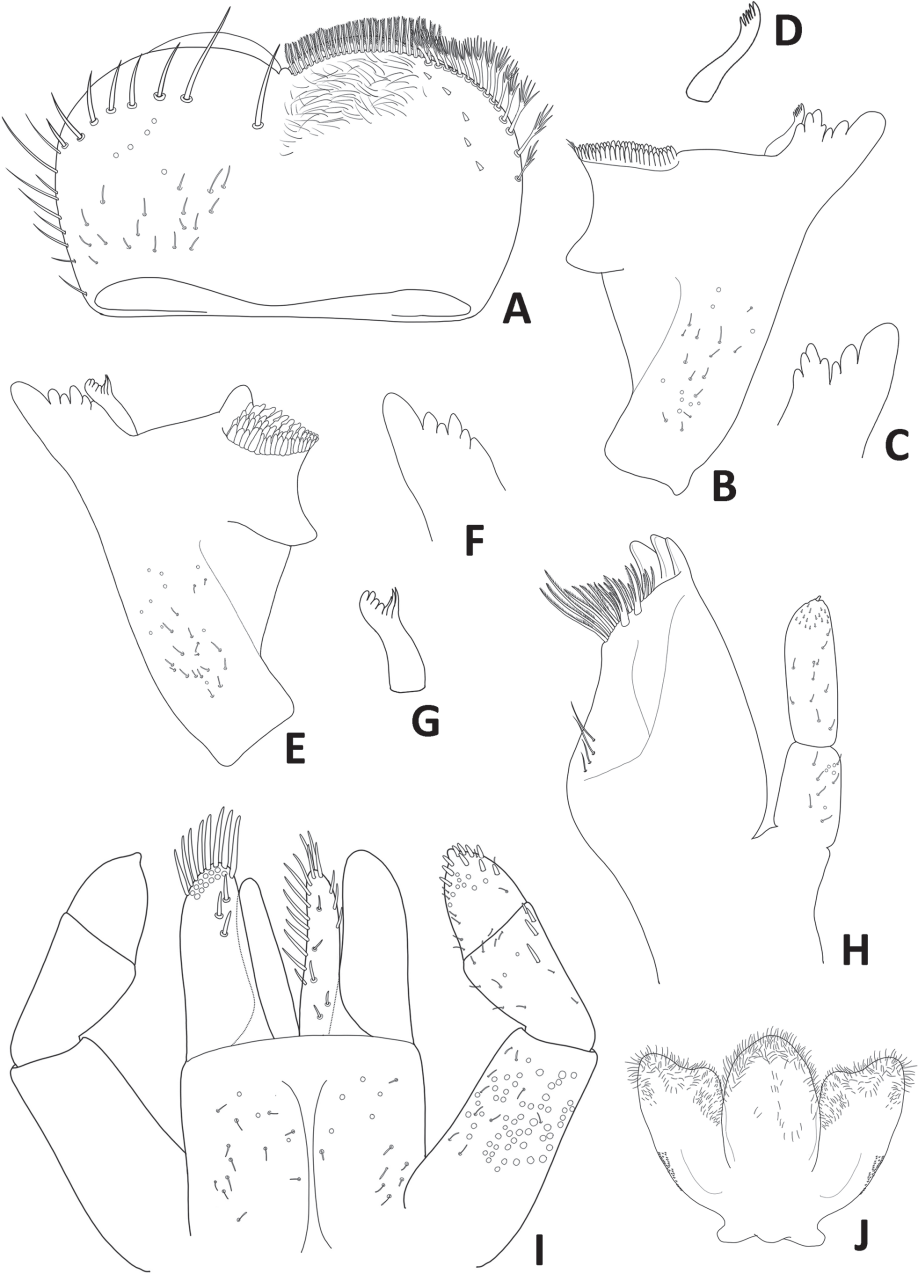
Baetiella (Baetiella) lannaensis sp. nov., larval morphology **A** labrum **B** right mandible **C** right incisors **D** right prostheca **E** left mandible **F** left incisors **H** left prostheca **G** left prostheca **H** maxilla **I** labium **J** hypopharynx.

**Figure 10.**
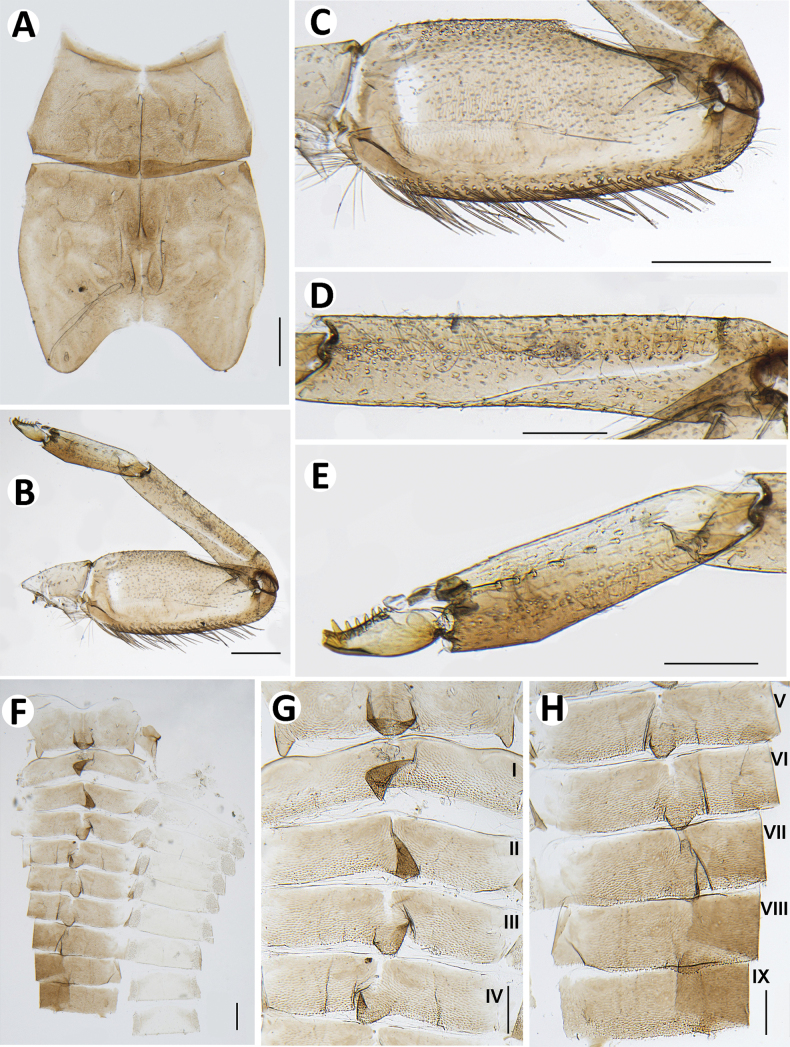
Baetiella (Baetiella) lannaensis sp. nov., larval morphology **A** pronotum and metanotum **B** foreleg **C** femur **D** tibia **E** tarsus and claw **F** abdomen **G** tergites I–IV **H** tergites V–IX. Scale bars: 0.2 mm (**A–E**); 0.1 mm (**F–H**).

**Figure 11.**
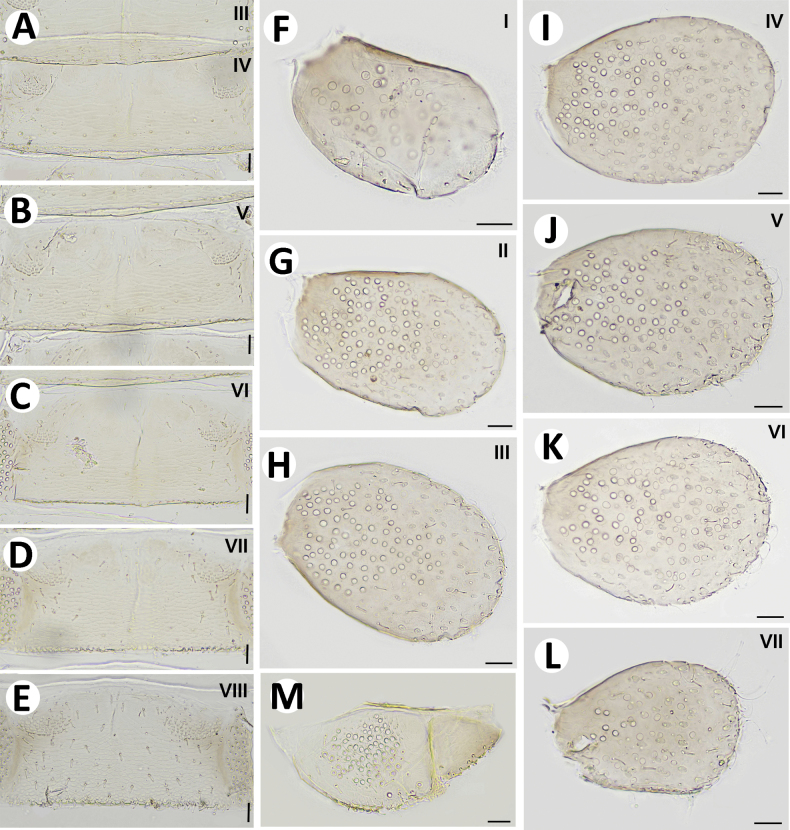
Baetiella (Baetiella) lannaensis sp. nov., larval morphology **A** sternites III and IV **B** sternites V **C** sternite VI **D** sternite VII **E** sternite VIII **F** gill I **G** gill II **H** gill III **I** gill IV **J** gill V **K** gill VI **L** gill VII **M** paraproct. Scale bars: 0.02 mm.

**Figure 12.**
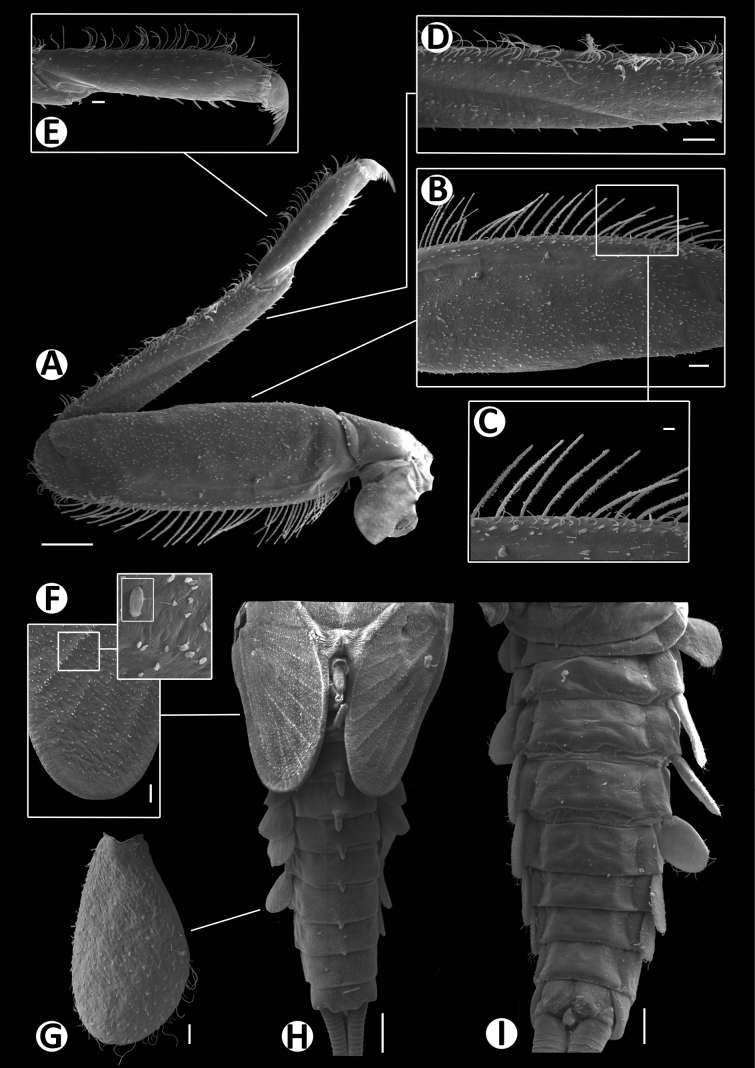
Baetiella (Baetiella) lannaensis sp. nov., larval morphology (SEM) **A** foreleg **B** femur **C** setae on dorsal margin of femur **D** tibia **E** tarsus and claw **F** forewing **G** gill VI **H** tergites **I** sternites. Scale bars: 100 µm (**A, I**); 30 µm (**B, C, D, F**); 20 µm (**E, G**); 200 µm (**H**).

**Figure 13.**
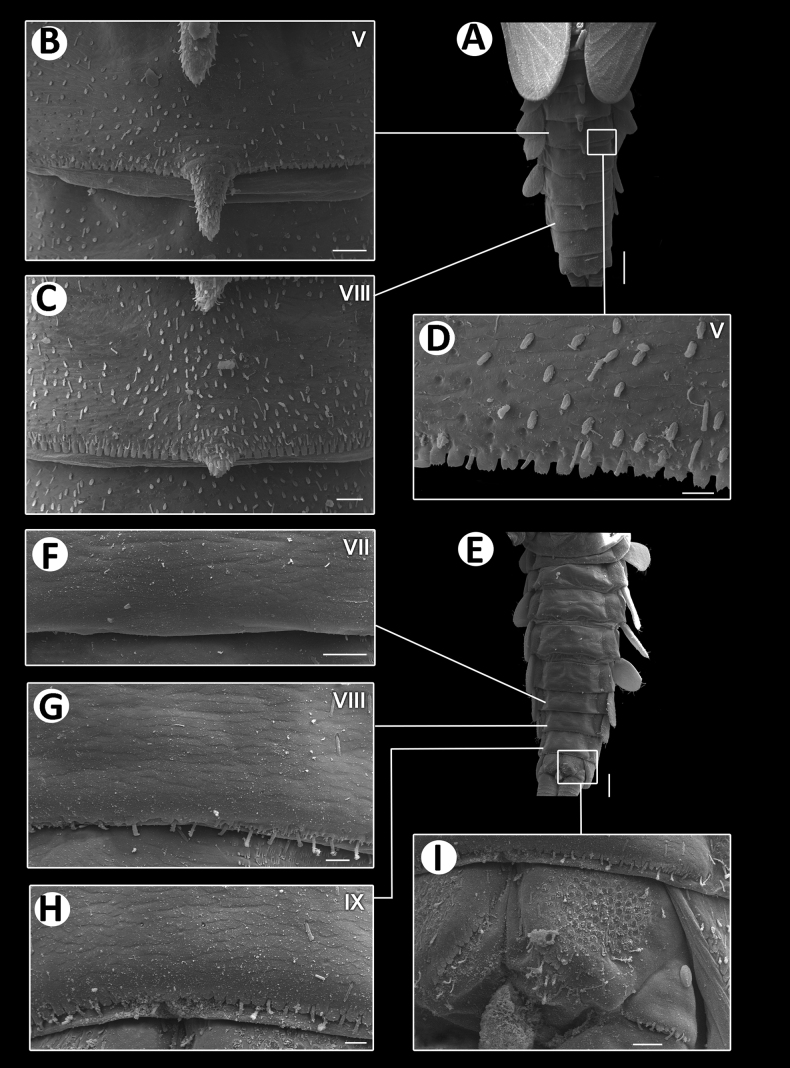
Baetiella (Baetiella) lannaensis sp. nov., larval morphology (SEM) **A** tergites **B** tergite V **C** tergite VIII **D** posterior margin of tergite V **E** sternites **F** sternite VII **G** sternite VIII **H** sternite IX **I** paraproct. Scale bars: 200 µm (**A**); 30 µm (**B**); 20 µm (**C, F, I**); 10 µm (**D, G, H**); 100 µm (**E**).

Figs 1 , 8 , 9 , 10 , 11 , 12 , 13

##### Type material examined.

***Holotype*.** Thailand, one larva on slide (KKU-AIC), Chiang Mai, Chom Thong district, Ban Luang, Siribhum waterfall, 18°32'50.02"N, 98°30'49.79"E, 1,359 m, 27.XII.2022, S. Phlai-ngam and B. Boonsoong leg. ***Paratypes*.** One larva on slide, 2 larvae on stubs, 3 larvae in alcohol, same data as holotype (KKU-AIC).

##### Other material examined.

Eight larvae in alcohol (KKU-AIC), Thailand, Mae Hong Son, Pai district, Mo Pang waterfall, 19°22'43.79"N, 98°22'32.87"E, 855 m, 10.V.2023, S. Phlai-ngam leg; two larvae in alcohol (KKU-AIC), Chiang Mai, Chom Thong District, Ban Luang, Siribhum waterfall, 18°32'50.02"N, 98°30'49.79"E, 1,359 m, 18.III.2023, B. Boonsoong leg.; 21 larvae in alcohol (KKU-AIC), Chiang Mai, Chom Thong district, Ban Luang, Siribhum waterfall, 18°32'50.02"N, 98°30'49.79"E, 1,359 m, 12.V.2023, S. Phlai-ngam leg. Two larvae in alcohol (MZL: GBIFCH01118451), Chiang Mai, Chom Thong district, Ban Luang, Siribhum waterfall, 18°32'50.02"N, 98°30'49.79"E, 1,359 m, 17.XII.2020, B. Boonsoong leg.

##### Diagnosis.

Baetiella (Baetiella) lannaensis sp. nov. differs from other species and can be identified by (i) tergites I–VIII with a single, reduced, posteromedian protuberance, which gradually diminishes in size towards the terminal segment; (ii) distal margin of tergites II–X with multi-dentated and blunt spines; (iii) distal margin of sternites VIII–X with multi-dentated, blunt denticles and scattered short, fine, simple setae along margin; (iv) dorsal surface of body and legs covered with numerous rounded scale-like setae; (v) gill surface with numerous rounded scales and short, fine setae; and (vi) dorsal margin of femur with a regular row of long, rounded, simple, ciliated setae; (vii) terminal segment of labial palp conical and symmetrical shaped with apical tip, segment II of labial palp with a small, reduced inner apical lobe.

##### Description.

Coloration (Figs 1A, B, 8A, B). Head dorsally brownish, with darker brown mark at frontal suture. Thorax dorsally brownish with dark brown pattern and with small dark brown tubercles. Abdominal tergites brownish with dark brown pattern (Fig. 8A); sternites pale brown (Fig. 8C). Legs brownish; dorsal surface brownish, with pale brown mark ventrally; dorsal surface of femur with pale brown longitudinal striped along dorsal margin distally, tarsus and claw distally darker brown. Caudal filaments brownish.

***Body*** dorsoventrally somewhat flattened (Fig. 8B). Paracercus reduced to one segment, cerci subequal to body length. Body surface covered with numerous rounded scale-like setae.

***Head*** ~2× wider than long.

Antenna (Fig. 8A). Length ~1.5× as long as head length; scape, pedicel and flagellum without process, without scale bases and spines, covered with scattered, fine setae; flagellum covered with scattered, fine setae in each segment.

***Mouthparts*.** Labrum (Fig. 9A). Broad, slightly rectangular; ~2× wider than long; each half of dorsal surface with one central seta and a row of seven long, simple, robust submarginal setae, proximal part with scattered, fine, simple setae; distal margin with anteromedian notch shallow, lateral margin with a row of long, fine, simple setae; ventral surface with a row of feathered setae along distal margin, distolateral margin with a row of feathered, setae and a row of five short, simple, robust setae near lateral margin, distal part with patch of dense, fine, hair-like setae.

Right mandible (Fig. 9B–D). Outer and inner incisors partially separated (Fig. 9B, C), incisors well developed, outer incisor with three denticles and inner incisor with four denticles; right prostheca (Fig. 9D) slender; edge between mola and prostheca smooth without spines; apex of mola with tuft of spine-like setae; proximal surface with scattered, short, fine, simple setae.

Left mandible (Fig. 9E–G). Outer and inner incisors almost completely fused (Fig. 9E, F), well developed incisors with six denticles apically, prostheca (Fig. 9G) robust, apically with small denticles and comb-shaped structure; edge between mola and prostheca smooth without spines; proximal surface with scattered, short, fine, simple setae.

Maxilla (Fig. 9H). Galea lacinia with three blunt, robust canines and a canine-like dentiseta; inner dorsal row of setae with two bifid pectinate dentisetae; inner ventral row of robust, simple pectinate setae medially, with long robust, simple pectinate setae distally; with a row of four long setae on ba­sis of galea lacinia. Maxillary palp 2-segmented, with scattered small, hair-like setae; distal segment with distinct, small tip at apex and small, hair-like setae.

Labium (Fig. 9I). Glossa basally broad, narrower toward apex, glossa slightly shorter than paraglossa, inner margin with a row of nine medium, stout, simple setae, apical margin with three or four long, stout, blunt, simple setae, ventral surface with scattered, short, fine setae; paraglossa sub-rectangular, broader than glossa, apical margin with three rows of medium, stout, simple setae and a row of medium, stout, simple setae on inner margin, dorsal surface with three medium, stout, simple setae subapically; labial palp 3-segmented, terminal segment conical and asymmetrical with small tip at apex; segment II with small, reduced inner apical lobe, distal part with three simple, robust setae near outer margin; ventral surface covered with scattered short, robust and fine setae; segments I and III with micropores.

Hypopharynx (Fig. 9J). Lingua rounded apically, with apical tuft of fine, long, simple setae; superlingua broadly truncate, covered with abundant, fine, simple setae.

***Thorax*** (Figs 8A, 10A) with numerous rounded scale-like setae; mesonotum with dorsal surface with two pairs of small, reduced, tubercles anteromedially and posteromedially; metanotum with a single, posteromedian protuberance.

Hindwing pad reduced, ~1.5× longer than width, covered with numerous round scale-like setae.

Legs (Fig. 10B–E). Forelegs. Femur (Figs 10C, 12B, C) length ~2.2× maximum width. Dorsal margin with row of long, rounded, simple, ciliated setae, length ~1/3 to 1/2 of femur width, decreasing at distal part. All dorsal and ventral margins with a scattered row of short, robust setae. Femoral villopore present. Median part of dorsal margin with scattered fine and dense rounded scale-like setae.

Tibia. Dorsal margin with a row of long, fine, simple setae (Figs 10D, 12D), both dorsal and ventral margins with a scattered row of short, robust setae, dorsal surface with numerous rounded scale-like (Fig. 12D).

Tarsus (Figs 10F, 12E) with a row of long, fine, simple setae dorsally, with a row of approximately seven robust, blunt setae increasing in size ventrally on distal part. Tarsal claw with a row of seven denticles with pair of subapical setae. All legs without coxal gill.

Midlegs and hindlegs as forelegs.

***Abdomen*** (Fig. 10F). Distal margin of tergites I–VIII with a single, posteromedian protuberance, decreasing in size.

Abdominal tergites (Figs 10G, H, 12H, 13A–D).

Distal margin of tergite I without denticles, surface with scattered, fine setae and rounded scale-like setae; distal margin of tergites II–X with multi-dentated and blunt spines, surface with scattered, fine setae and numerous rounded scale-like setae; tergites IX–X without posteromedian protuberance.

Abdominal sternites (Figs 11A–E, 12I, 13E–H). Distal margin of sternites I–VII smooth without denticles or scales-like setae, surface with scattered short, fine, simple setae (Figs 11A–C, 12I, 13E, F); distal margin of sternites VIII–X with multi-dentated, blunt denticles and scattered short, fine, simple setae along margin, surface with scattered short, fine, simple setae (Figs 11D, E, 12I, 13G, H).

Gills (Figs 11F–L, 12G). Seven pairs of gills present on abdominal tergites I–VII (Fig. 11F–L), oval and without tracheation; gill I smaller than other gills, coloration reddish to brown medially with translucent on outer margin, gill surface with scattered, fine setae and several rounded scales and micropores, gill margin with scattered hair-like setae (Fig. 12G).

Paraproct (Figs 11M, 13I) with numerous micropores and scattered short, fine setae medially, inner margin with 12–14 serrations and multi-dentate, blunt denticles along margin.

**Imago.** Unknown.

##### Ecological notes.

The larvae of Baetiella (Baetiella) lannaensis sp. nov. were collected in Siribhum and Mo Pang waterfalls, in the same microhabitats and substrate types than Baetiella (Baetiella) baei sp. nov.; they were found in boulders substrates with fast-flowing water. However, this species was more abundant than Baetiella (Baetiella) baei sp. nov. (Fig. 23).

##### Etymology.

This specific epithet, *lannaensis*, refers to the Lanna kingdom, the historic name of northern Thailand where this new species was found.

##### Distribution.

Chiang Mai and Mae Hong Son Provinces (northern Thailand).

##### Remarks.

The newly discovered species is morphologically similar to *Baetiellaausobskyi* Braasch, 1983; both species share tergites I–VIII with a single, reduced, posteromedian protuberance and dorsal surface of labrum with a row of fewer than seven submarginal setae. Nevertheless, Baetiella (Baetiella) lannaensis sp. nov. reveals distinct characters that distinguish it from *B.ausobskyi*. For instance, the dorsal margin of femur with a row of long, rounded, simple, feathered setae while this row is compound of long, robust, rounded, simple setae in *B.ausobskyi*. This new species can be identified by the surface of body, legs, and especially the gill surface that are covered by numerous rounded scale-like setae. The distal margin of sternites VIII–X of Baetiella (Baetiella) lannaensis sp. nov. possesses multi-dentated and blunt spines. This new species exhibits a reduction of the paracercus to a single segment, whereas *B.ausobskyi*, as described by Braasch (1983), has a reduction to three segments. The two species also differ by the shape of the inner apical lobe of segment II of the labial palp; this lobe is more reduced in Baetiella (Baetiella) lannaensis. Additionally, the terminal segment of the labial palp in Baetiella (Baetiella) lannaensis sp. nov. is conical and asymmetrical, whereas in *B.ausobskyi*, this segment is rounded, conical, and nearly symmetrical (Vasanth et al. 2020).

#### ﻿ Baetiella (Baetiella) bibranchia sp. nov.

Taxon classificationAnimaliaEphemeropteraBaetidae

427B056A-5F29-5194-835A-F58A95FEEA6B

https://zoobank.org/B6E6792D-F757-4998-A935-18ADD2EA6BF1

**Figure 14.**
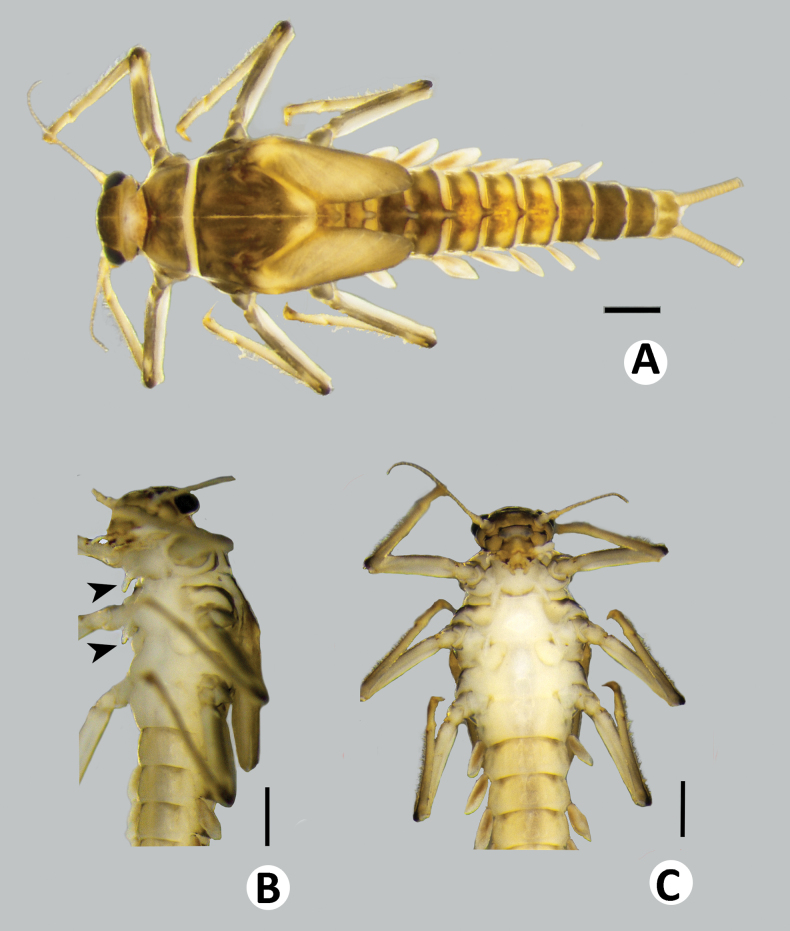
Baetiella (Baetiella) bibranchia sp. nov., larval habitus (paratype) **A** dorsal view **B** lateral view (black arrow indicating coxal gills) **C** ventral view. Scale bars: 1 mm.

**Figure 15.**
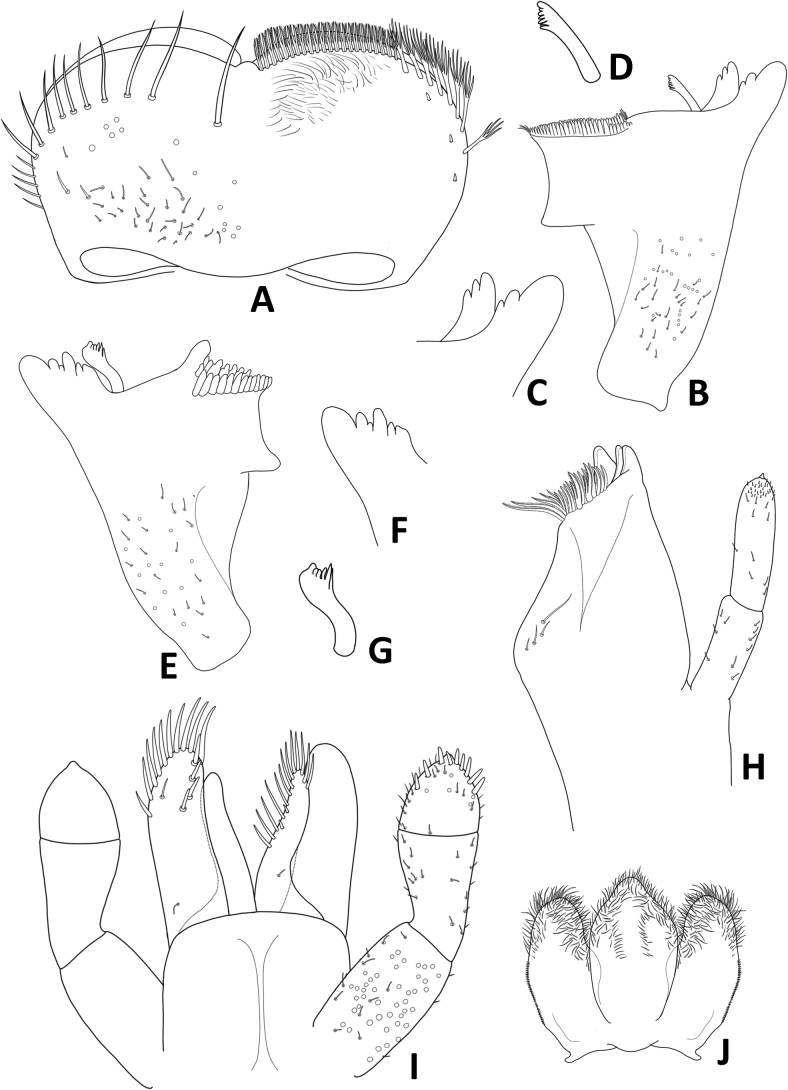
Baetiella (Baetiella) bibranchia sp. nov., larval morphology **A** labrum **B** right mandible **C** right incisors **D** right prostheca **E** left mandible **F** left incisors **H** left prostheca **G** left prostheca **H** maxilla **I** labium **J** hypopharynx.

**Figure 16.**
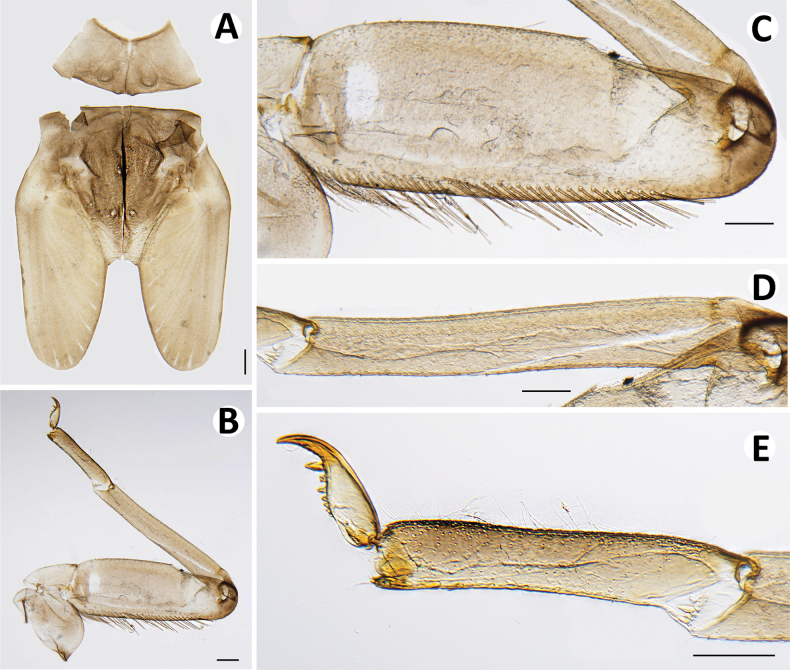
Baetiella (Baetiella) bibranchia sp. nov., larval morphology **A** pronotum and metanotum **B** foreleg **C** femur **D** tibia **E** tarsus and claw. Scale bars: 0.5 mm (**A**); 0.2 mm (**B**); 0.1 mm (**C, D, E**).

**Figure 17.**
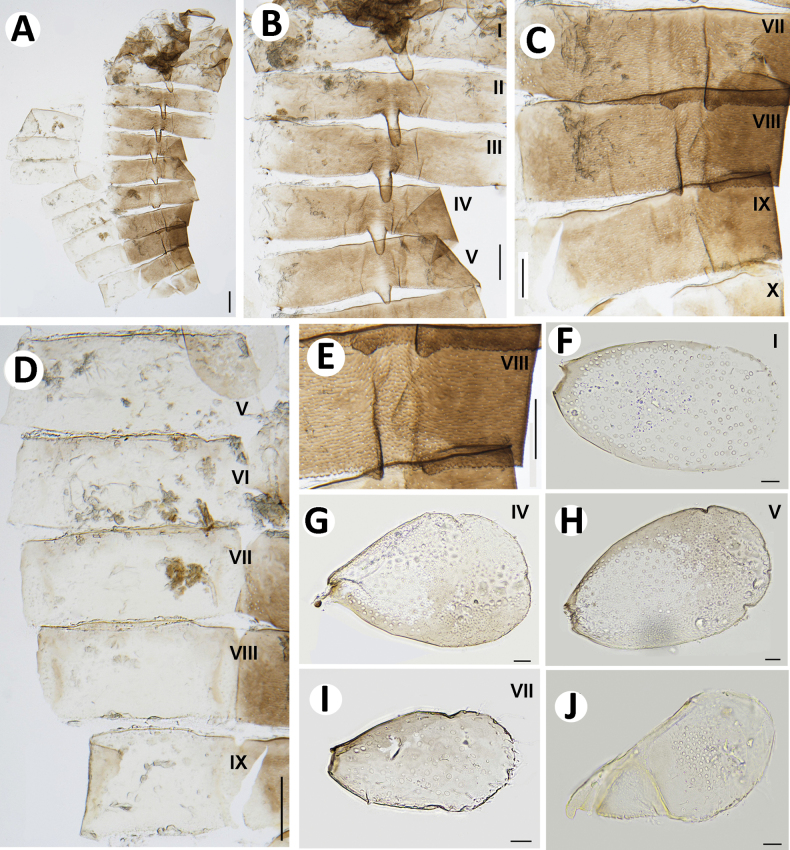
Baetiella (Baetiella) bibranchia sp. nov., larval morphology **A** abdomen **B** tergites I–V **C** tergites VII–X **D** sternites V–IX **E** distal margin of tergite VIII **F** gill I **G** gill IV **H** gill V **I** gill VII **J** paraproct. Scale bars: 0.2 mm (**A, B, D**); 0.1 mm (**C, E**); 0.02 mm (**I, J**).

**Figure 18.**
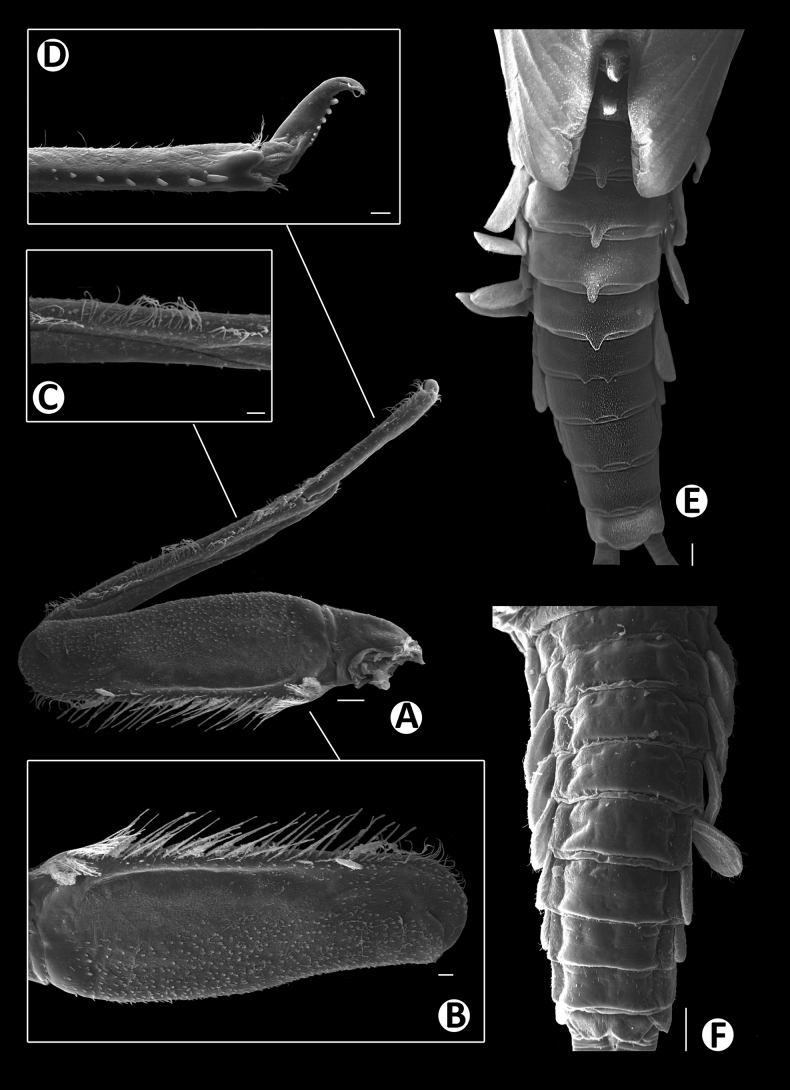
Baetiella (Baetiella) bibranchia sp. nov., larval morphology (SEM) **A** foreleg **B** femur **C** tibia **D** tarsus and claw **E** tergites **F** sternites. Scale bars: 20 µm (**A–D**); 100 µm (**E, F**).

**Figure 19.**
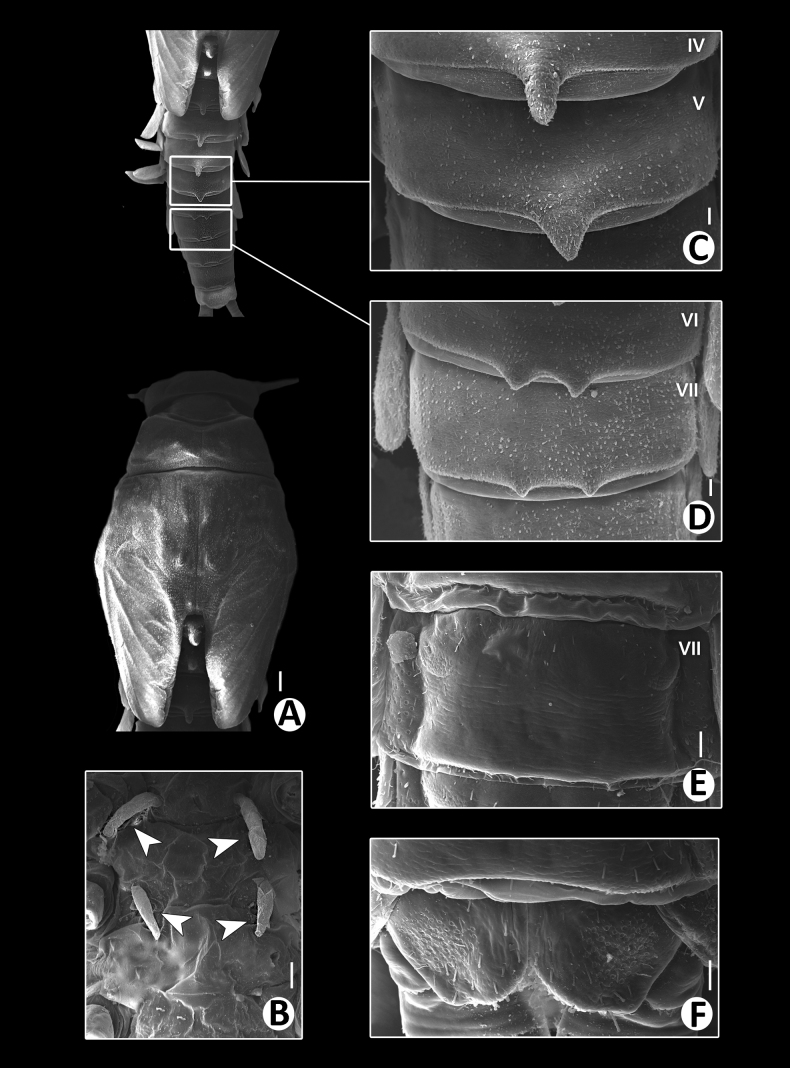
Baetiella (Baetiella) bibranchia sp. nov., larval morphology (SEM) **A** thorax (Dorsal) **B** thorax (Ventral) (White arrows pointed coxal gills) **C** tergites IV–V **D** tergites VI–VII **E** sternite VII **F** paraproct. Scale bars: 20 µm (**A–D**); 100 µm (**E, F**).

Figs 14 , 15 , 16 , 17 , 18 , 19

##### Type material examined.

***Holotype*.** Thailand, One larva on slide (KKU-AIC), Chiang Rai, Muang district, Pong Phra Baht waterfall, 20°00'39.60"N, 99°48'14.47"E, 476 m, 11.III.2021, B. Boonsoong leg. ***Paratypes*.** Two larvae on stubs, Six larvae in alcohol, same data as holotype (KKU-AIC); Four larvae in alcohol, same data as holotype (MZL: GBIFCH01118452).

##### Diagnosis.

This new species, Baetiella (Baetiella) bibranchia sp. nov. can be easily distinguished from other *Baetiella* species by the following combination of characters; (i) distal margin of tergites I–V with a single, posteromedian protuberance, distal margin of tergites VI–IX with a pair of posteromedian protuberances, the distance between posteromedian protuberances gradually widened backwards, distance between bases of posteromedian protuberances of tergite VI is shorter than length of posteromedian protuberances; (ii) coxal gills present at the base of forelegs and midlegs; (iii) terminal segment reduced to one segment; (iv) segment II of labial palp without inner apical lobe; (v) distal margin of tergites II–X with muti-dentated, blunt denticles; (vi) distal margin of sternites smooth without denticles.

##### Description.

Coloration (Fig. 14A). Head dorsally brownish, with darker brown mark at frontal suture. Thorax dorsally brownish yellow with dark brown pattern and with very small dark brown tubercles. Abdominal tergites brownish with darker brown pattern; sternites pale brown (Fig. 14B, C). Legs brownish; dorsal surface brownish, pale brown ventrally; dorsal surface of femur with pale brown marking distally, tarsus and claw distally dark brown. Caudal filaments brownish.

***Body*.** Dorsoventrally somewhat flattened (Fig. 14B, C). Paracercus reduced to one segment, cerci subequal to body length. Body surface covered with scattered rounded scale-like setae.

***Head*** ~2× wider than long.

Antenna. Length ~1.5× as long as head length; scape, pedicel and flagellum without process, without scale bases and spines, covered with scattered, fine setae; flagellum covered with scattered, fine setae in each segment.

***Mouthparts*.** Labrum (Fig. 15A). Broad, slightly rectangular; ~2× wider than long; each half of dorsal surface with one central seta and a row of nine long, simple, robust submarginal setae, proximal part with scattered, fine, simple setae; distal margin with anteromedian notch shallow, lateral margin with a row of medium, fine, pointed, simple setae; ventral surface with a row of feath­ered setae along distal margin, distolateral margin with a row of feathered setae and a row of three, short, simple, robust setae near lateral margin, distal part with patch of dense, fine, hair-like setae.

Right mandible (Fig. 15B–D). Outer and inner incisors partially separated with visible separating line (Fig. 15B, C), incisors well developed, outer incisor with three denticles and inner incisor with three denticles; right prostheca (Fig. 15D) slender with denticles apically; edge between mola and prostheca smooth without spines; apex of mola with tuft of spine-like setae; proximal surface with scattered short, fine, simple setae.

Left mandible (Fig. 15E–G). Outer and inner incisors almost completely fused (Fig. 15E, F), well developed incisors with six denticles apically, prostheca robust, apically with small denticles and comb-shaped structure (Fig. 15G); edge between mola and prostheca smooth without spines; proximal surface with scattered short, fine, simple setae.

Maxilla (Fig. 15H). Galea lacinia with three blunt, robust canines and a canine-like dentiseta; inner dorsal row of setae with two bifid pectinate dentisetae; inner ventral row of robust, simple pectinate setae medially, with long robust, simple pectinate setae distally; basal with a row of four long basal setae on basis of galea lacinia. Maxillary palp 2-segmented, with scattered small, hair-like setae; distal segment with distinct, small tip at apex and small, hair-like setae.

Labium (Fig. 15I). Glossa basally broad, narrower toward apex, glossa slightly shorter than paraglossa, inner margin with a row of eight medium, stout, simple setae and two short, stout, simple setae, apical margin with four or five long, stout, simple setae, proximal part with a short, fine, robust seta; paraglossa sub-rectangular, broader than glossa, apical margin with three rows of long, stout, simple setae and a row of stout setae on outer margin, dorsal surface with four median, stout, simple setae and a long, fine, simple seta apically, proximal part with a short, fine seta. Labial palp 3-segmented, terminal segment conical, rounded and asymmetrical with small tip at apex; segment II without inner apical lobe; ventral surface covered with scattered short, robust, simple setae and fine setae; segments I and III with numerous micropores.

Hypopharynx (Fig. 15J). Lingua rounded apically, with apical tuft of fine, long simple setae; superlingua apically rounded, covered with abundant fine simple setae.

***Thorax*** (Figs 16A, 19A) with a very small, reduced tubercle. Metanotum with a single posteromedian protuberance

Hindwing pad reduced, ~1.5× longer than width, covered with numerous rounded, scale-like setae.

Legs (Figs 16B–E, 18A–D). Forelegs. Femur. Length ~3× maximum width. Dorsal margin with a row of long, rounded, simple setae, ~1/3 of femur width, decreasing at distal part (Figs 16C, 18B). Dorsal and ventral margins with a scattered row of short, robust setae. Femoral villopore present. Medial part of dorsal surface with scattered fine and rounded scale-like setae.

Tibia with a row of long, fine, simple setae dorsally (Figs 16D, 18C), dorsal and ventral margins with a row of short, robust setae, dorsal surface with scattered, short, fine setae.

Tarsus with a row of long, fine, simple setae dorsally, with a row of approximately seven robust, blunt setae ventrally increasing in size on distal part. Tarsal claw with a row of seven denticles with pair of subapical setae (Figs 16E, 18D).

Membranous digitiform extra gills at base of coxa (Figs 14B, C, 19B).

Midlegs and hindlegs as forelegs, except extra gills absent at base of coxa of hindlegs.

***Abdomen*** (Fig. 17A). Distal margin of tergite I–V with a single, posteromedian protuberance, distal margin of tergites VI–IX with a pair of posteromedian posteromedian protuberances.

Abdominal tergite I with distal margin smooth, without denticles; distal margin of tergites II–X with multi-dentated, blunt denticles along margin (Figs 17C, E, 18E, 19C, D). Surface of all tergites covered with scattered short, fine, simple setae and rounded scale-like setae.

Abdominal sternites (Figs 17D, 18F). Sternites with distal margin smooth without denticles or scale-like setae, surface with scattered short, fine, simple setae (Fig. 19E).

Gills (Fig. 17F–I). Seven pairs of gills present on abdominal tergites I–VII, oval and without tracheation; gill VII smaller than other gills (Fig. 17I), coloration reddish to brown, small area medially translucent on outer margin; gill surface with scattered fine setae and numerous micropores, margin with scattered hair-like setae (Fig. 17F–I).

Paraproct (Figs 17J, 19F). Surface with numerous micropores and scattered short, fine setae; inner and outer margins with several serrations and multi-dentated, blunt denticles along margin.

**Imago.** Unknown.

##### Ecological notes.

The larvae were collected in Pong Phra Baht waterfall in northern Thailand (Chiang Rai Province). The larvae were found in headwater streams with intact forest canopy in mountainous areas at medium altitude (~780 m a.s.l.). The larvae were found on surface of boulders in fast-flowing water like the other new species.

##### Etymology.

This specific epithet, *bibranchia*, is combination of *bi*- in reference to two and -*branchia* in reference to gills. The name *bibranchia* highlights the remarkable presence of two pairs of coxal gills, an important diagnostic character of this new species.

##### Distribution.

Chiang Rai Provinces (Northern Thailand).

##### Remarks.

Baetiella (Baetiella) bibranchia sp. nov. presents similarities with other *Baetiella* species including *Baetiellabispinosa* (Gose, 1980), *Baetiellatrispinata* Tong & Dudgeon, 2000, *Baetiellamacani* (Müller-Liebenau, 1985), and *Baetiellasubansiri* Vasanth, Selvakumar & Subramanian, 2020. These species present single protuberances on the first tergites and paired posteromedian protuberances on the following tergites (Table 4). Combined with presence/absence of coxal gills, the number of tergites with paired or unpaired protuberances is a reliable character for separating species. Both *Baetiellabispinosa* and *B.macani* possess paired posteromedian protuberances on tergites III–V and all of their legs have coxal gills. They can be separated by the number of gills as *B.bispinosa* possesses a total of seven pairs and *B.macani* only six. *Baetiellatrispinata* can be distinguished from the aforementioned species by the complete absence of coxal gills. Beside possessing finger-like coxal gills on all of the legs, *Baetiellasubansiri* presents distinct morphological characters, particularly elongated abdominal gills and the presence of more developed posteromedian protuberances.

#### ﻿ Baetiella (Baetiella) bispinosa

Taxon classificationAnimaliaEphemeropteraBaetidae

(Gose, 1980)

DEFEB2F3-7F39-526D-94E8-8AD8E4962384

Fig. 20

Pseudocloeon bispinosus Gose, 1980: 211
Baetiella bispinosa : Waltz and McCafferty 1987: 563; Tong and Dudgeon 2000: 143

##### Material examined.

Thailand, Six larvae in alcohol (KKU-AIC), Chiang Rai, Phan district, stream near Pha Khong cave, 19°31'12.15"N, 99°39'12.59"E, 649 m, 5.II.2016, S. Phlai-ngam leg.; Five larvae in alcohol (KKU-AIC), Chiang Mai, Mae Taeng district, Mae Taeng Elephant Kraal, Mae Taeng River, 19°11'50.51"N, 98°53'13.98"E, 362 m, 5.I.2007, N. Tungpairojwong leg. Three larvae in alcohol (MZL: GBIFCH01118453), Chiang Rai, Mae Chan district, Nang Lae Nai waterfall, 20.084703°N, 99.732234°E, 470 m, 7.V.2019, B. Boonsoong leg.

##### Diagnosis.

**Figure 20.**
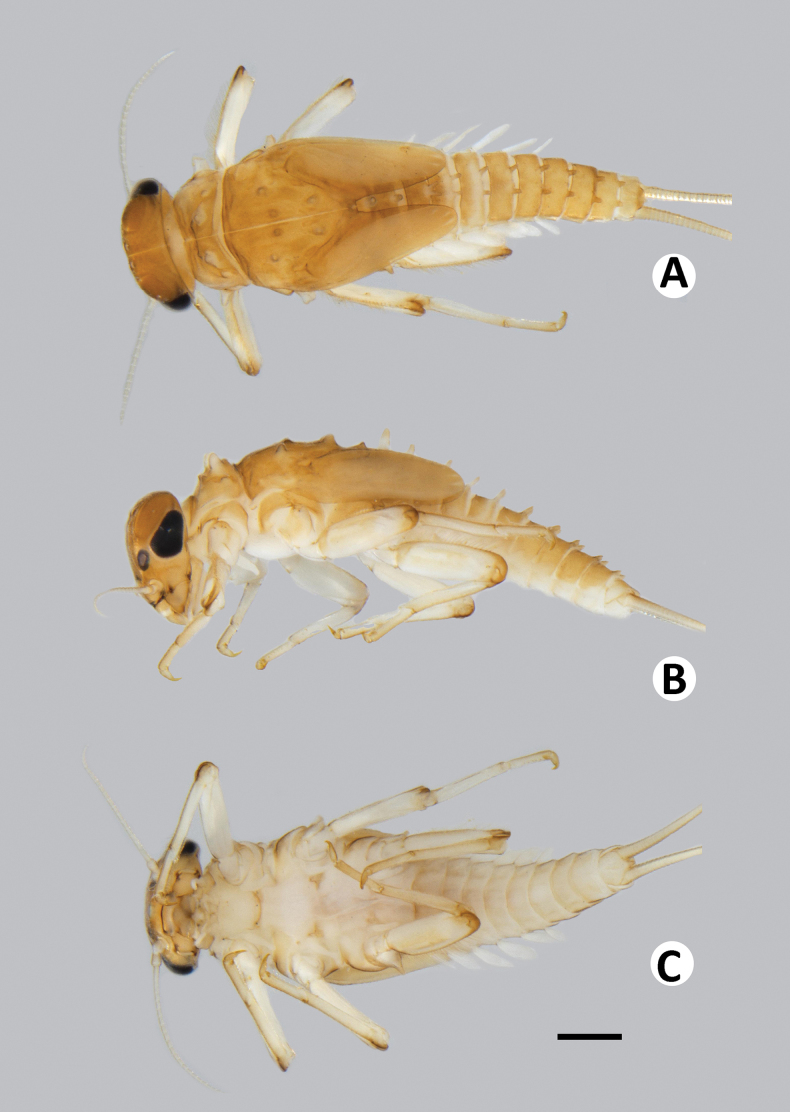
Baetiella (Baetiella) bispinosa, larval habitus **A** dorsal view **B** lateral view **C** ventral view. Scale bar: 1 mm.

Body (Fig. 20A–C) dorsoventrally flattened, brownish with darker pattern, head and thorax dorsally brownish; maxillary palp 2-segmented, subequal in length, terminal segment with a small tip at apex; labial palp 3-segmented, terminal segment conical with a small tip at apex, segment II with a small, reduced inner apical lobe; thorax with distinct tubercles, pronotum with two pairs of tubercles medially, mesonotum with a pair of tubercles medially and with two pairs sub-medially; posterior margin of metanotum with a single posteromedian tubercle; hindwing pads vestigial; all legs with a single finger-like coxal gills, femora with a row of long, dense, fine, glabrate setae on dorsal margin, tarsal claw with two rows of denticles and a pair of apical setae; distal margin of tergites I and II with a single posteromedian protuberance, tergites III–IX with a pair of posteromedian protuberances, the distance between posteromedian protuberances gradually widened backwards; tergal surface with numerous micropores and short, fine, simple setae, distal margin with blunt denticles; seven pairs of gills present on abdominal tergites I–VII, oval and without tracheation; paracercus reduced to one segment.

##### Distribution.

Chiang Rai and Chiang Mai Provinces (northern Thailand).

##### Remarks.

The larvae of B. (Baetiella) bispinosa were collected on the surface of cobbles and boulders substrates in moderate to fast flowing streams. We found this species only in headwater streams.

#### ﻿ Baetiella (Gratia) narumonae

Taxon classificationAnimaliaEphemeropteraBaetidae

(Boonsoong & Thomas, 2004)

6F8B43CF-B585-5168-8F0D-0D3B25B4BB5C

Fig. 21

Gratia narumonae Boonsoong & Thomas, 2004: 1.
Baetiella narumonae : Kluge 2022: 166.

##### Material examined.

Thailand, Six larvae in alcohol (KKU-AIC), Nan, Bo Kluea district, 18°59'47.31"N, 101°12'50.94"E, 684 m, 24.XII.2019, S. Phlai-ngam leg.; Ten larvae in alcohol (KKU-AIC), Chiang Mai, Chom Thong district, near Mae Ya waterfall, 18°26'22.44"N, 98°35'51.77"E, 582 m, 3.II.2016, S. Phlai-ngam leg.; Three larvae in alcohol (KKU-AIC), Chiang Mai, Chom Thong district, Mae Klang stream, 18°29'39.72"N, 98°40'06.65"E, 337 m, 3.II.2016, S. Phlai-ngam leg. Two larvae in alcohol (MZL: GBIFCH01118454), Chiang Mai, Chom Thong district, Ban Luang, Siribhum waterfall, 18°32'50.02"N, 98°30'49.79"E, 1,359 m, 17.XII.2020, B. Boonsoong leg. Four larvae in alcohol (MZL: GBIFCH01118455), Chiang Rai, Mae Lao district, Khun Korn waterfall, 19°51.768'N, 99°39.078'E, 534 m, 6.V.2019, B. Boonsoong leg.

##### Diagnosis.

**Figure 21.**
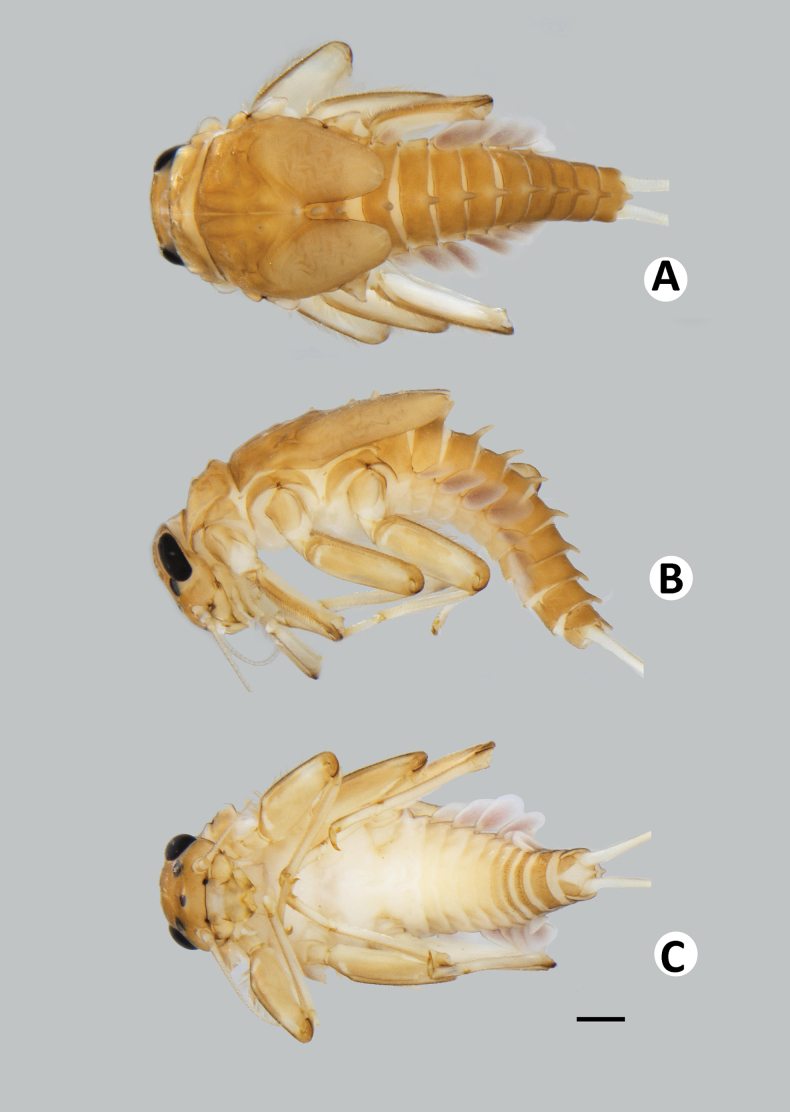
Baetiella (Gratia) narumonae, larval habitus **A** dorsal view **B** lateral view **C** ventral view. Scale bar: 1 mm.

Body (Fig. 21A–C) dorsoventrally flattened, brownish with dark brown pattern, head and thorax dorsally brownish, thorax broad; labrum broad (slightly broader than labium), each half of dorsal surface with one central seta, distal part with a row of submarginal setae including two innermost long, pointed, simple, robust setae and others with branched, robust setae; maxillary palp 2-segmented, short and thick, subequal in length, terminal segment with a small tip at apex; labium compact, glossa clearly shorter than paraglossa; labial palp 3-segmented, terminal segment rounded with a small tip at apex, segment II with an inner apical lobe; thorax with distinct tubercles, pronotum with two pairs of tubercles, the anterior pair bigger and at wider interval than posterior ones; hindwing pad reduced; all legs without coxal gill, femora with a strong row of long, dense, ciliated setae on dorsal margin, tarsal claw with a row of 8–10 denticles and a pair of apical setae; dorsal surface shagreened, with short, rounded scale-like setae and scattered, fine setae; tergites I–IX with a strong, single posteromedian protuberance and reduced to small size in tergite X, distal margin with short, blunt denticles and scattered, short, fine setae; sternites with rounded, scale-like setae and scattered, short, fine setae, sternite VI with more abundant and bigger scales, sternites VII–IX with abundant scale-like setae, more numerous than in other segments, especially distal part and distal margin; seven pairs of gills present on abdominal tergites I–VII, oval and without tracheation, gill margin devoid of scales, only with scattered, fine setae; paraproct with 11–16 strong, rounded scale-like setae; paracercus reduced, cone-shaped.

##### Distribution.

Chiang Mai and Nan provinces (northern Thailand).

##### Remarks.

The larvae of Baetiella (Gratia) narumonae were found in fast flowing streams. The substrate types were dominated by boulders, cobbles, pebbles, gravel, and a sandy bottom. The larvae were found on the surface of boulders, cobbles, and bedrock, sometimes covered by green algae. Almost all sampling sites were in mountainous forest areas in the northern part of Thailand, undisturbed by human activities. However, this species can be also found in streams such as Mae Klang stream which can be disturbed by tourist attractions and human activities.

#### ﻿ Baetiella (Gratia) sororculaenadinae

Taxon classificationAnimaliaEphemeropteraBaetidae

(Thomas, 1992)

BA452440-9A30-5627-81D4-9EAA91C4EF34

Fig. 22

Gratia sororculaenadinae Thomas, 1992: 47.

##### Material examined.

Thailand, One larva in alcohol (KKU-AIC), Mukdahan, Nong Sung district, Tad Ton waterfall, 16°29'40.48"N, 104°18'47.09"E, 208 m, 23.XII.2017, S. Phlai-ngam leg; Four larvae in alcohol (KKU-AIC), Chiang Mai, Muang district, Suthep subdistrict, near Mon Tha Than waterfall, 18°49'02.41"N, 98°55'23.23"E, 713 m, 4.II.2016, S. Phlai-ngam leg.

##### Diagnosis.

**Figure 22.**
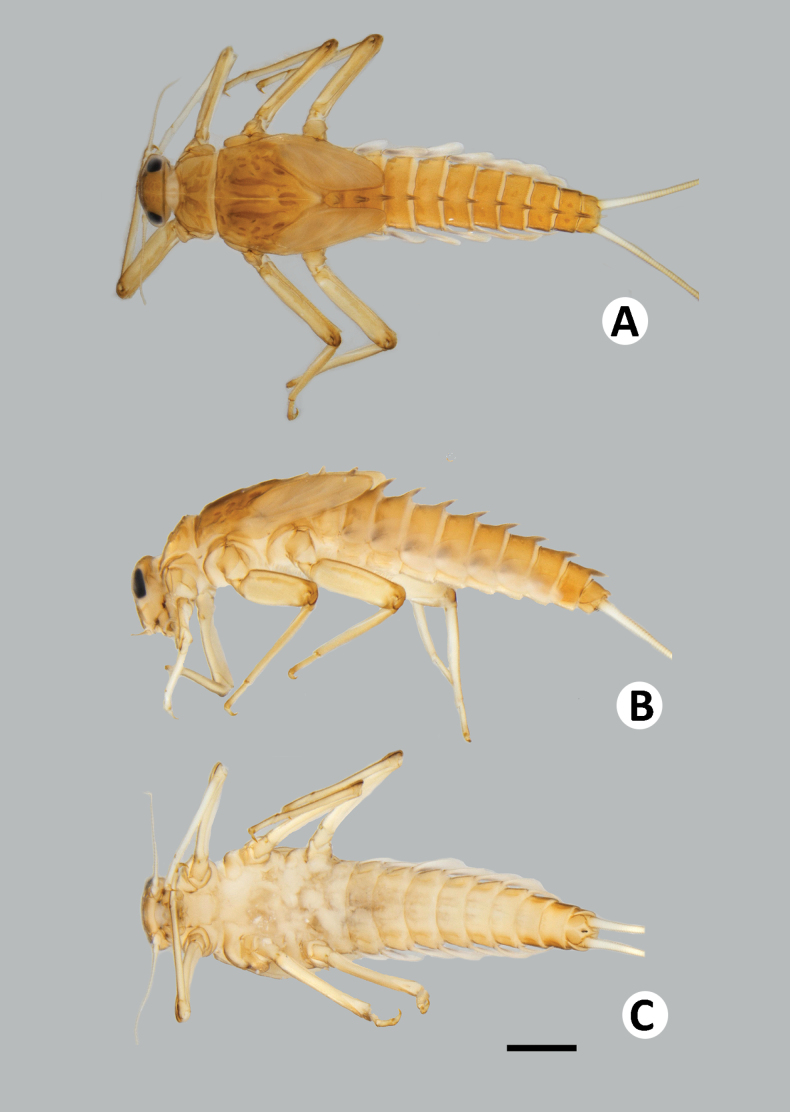
Baetiella (Gratia) sororculaenadinae, larval habitus **A** dorsal view **B** lateral view **C** ventral view. Scale bar: 1 mm.

Body (Fig. 22A–C) dorsoventrally flattened, brownish with dark brown pattern, head and thorax dorsally brownish, scape and pedicel with numerous scale-like setae; thorax broad; labrum broad, each half of dorsal surface with one central seta, distal part with a row of submarginal setae including two innermost long, pointed, simple, robust setae and others with branched, clearly fimbriate and ramified setae; maxillary palp 2-segmented, terminal segment with a small tip at apex; labium compact, glossa subequal in length to paraglossa; labial palp 3-segmented, terminal and segment II completed fused, terminal segment asymmetrical and rounded with a small tip at apex, segment II without an inner apical lobe; hindwing pad reduced; all legs without coxal gill, femora with a strong row of long, dense, ciliated setae on dorsal margin, tarsal claw with a row of 7–9 denticles and a pair of apical setae; tergites I–IX with a strong, single posteromedian protuberance, distal margin with short, blunt denticles and scattered, short, fine setae; seven pairs of gills present on abdominal tergites I–VII, oval and without tracheation, gill margins with numerous spine like-setae and scattered, short, fine setae; paraproct with 20–25 strong, rounded scales; paracercus reduced to one segment.

**Figure 23.**
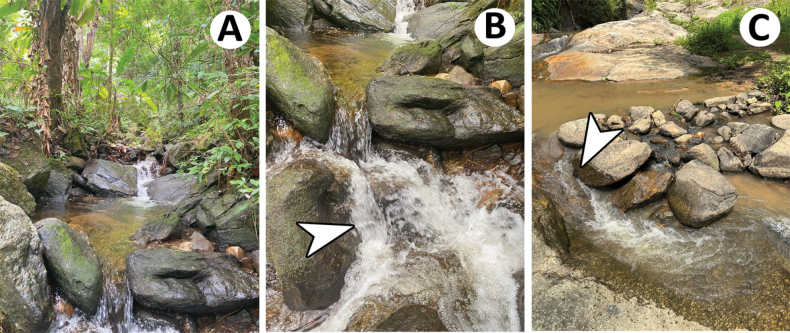
Sampling sites and the coexistence microhabitats of Baetiella (Baetiella) baei sp. nov. and B. (Baetiella) lannaensis sp. nov. **A** Siribhum waterfall (Chiang Mai province) **B, C** microhabitats of Siribhum waterfall and Mo Pang waterfall (Mae Hong Son Province) respectively. White arrows indicate coexisting microhabitats of B. (Baetiella) baei sp. nov. and B. (Baetiella) lannaensis sp. nov.

**Figure 24.**
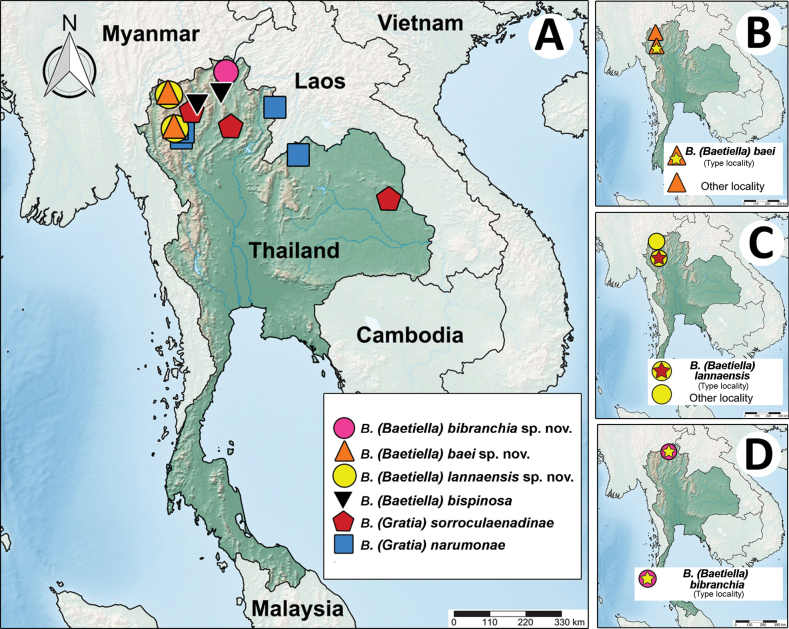
Distribution map of *Baetiella* species in Thailand with type localities of the new species. Red and yellow stars show the type localities.

##### Distribution.

Chiang Mai and Mukdahan Provinces (north and northeast of Thailand).

##### Remarks.

The larvae of Baetiella (Gratia) sororculaenadinae were found in fast flowing streams in forest areas. The substrate types were dominated by bedrock and boulders. The larvae were found on the surfaces of the bedrock. This species was reported from Chiang Mai Province (northern region of Thailand) for the first time by Thomas (1992). Interestingly, we found larvae of this species in the Mukdahan Province located in the northeast region of Thailand. This discovery indicates that this species is more widespread and colonizes wider environmental and altitudinal ranges than previously thought.

### ﻿Key to species of the mature larvae of the genus *Baetiella* in Thailand

**Table:** 

1	Submarginal setae on labrum branched and feathered; dorsal margin of femur with a regular row of setae	**Baetiella (Gratia) 2**
–	Simple submarginal setae on labrum (Figs 3A, 9A, 15A); dorsal margin of femur with irregular or regular row of setae	**Baetiella (Baetiella) 3**
2	Posteromedian protuberances present on abdominal tergites I–IX; labial palp segment II without inner apical lobe	**B. (Gratia) sororculaenadinae**
–	Posteromedian protuberances present on abdominal tergites I–X; labial palp segment II with an inner apical lobe	**B. (Gratia) narumonae**
3	Coxal gills present (Figs 14B, 19B)	**4**
–	Coxal gills absent	**5**
4	Tergites I and II with single, posteromedian protuberance; tergites III–IX with a pair of posteromedian protuberances (Fig. 20A); coxal gills present on all legs	**B. (Baetiella) bispinosa**
–	Tergites I–V with single, posteromedian protuberance; tergites VI–IX with a pair of posteromedian protuberances (Figs 14A, 18E); coxal gills present on forelegs and midlegs only	**B. (Baetiella) bibranchia sp. nov**.
5	Single, posteromedian protuberance present on tergites I–VIII (Figs 8A, 12H); dorsal margin of femur with a regular row of long, rounded, simple, ciliated setae (Fig. 12B, C)	**B. (Baetiella) lannaensis sp. nov.**
–	Single, reduced posteromedian protuberance present only on tergite I–III (Figs 2A, 4H); dorsal margin of femur with dense irregular row of long, fine, simple setae (Figs 4C, 6B, C)	**B. (Baetiella) baei sp. nov**.

### ﻿Molecular analysis

#### ﻿Pairwise genetic distance and reconstruction phylogenetic tree

COI sequences have been obtained from nine specimens collected in two localities (provinces of Chiang Mai and Chiang Rai). The newly obtained sequences were analyzed and compared with sequences sourced from databases such as GenBank and BOLD. The new sequences were submitted to the GenBank database, and their accession numbers are shown in Table 2.

The K2P analysis, which was conducted to evaluate genetic distances, shows that the three populations of the newly discovered species have low intraspecific variation, ranging from 0% to 2%. Baetiella (Baetiella) lannaensis sp. nov. has the lowest level of intraspecific genetic variation while B. (Baetiella) baei sp. nov. and B. (Baetiella) bibranchia sp. nov. have intraspecific distances of 1% and 2%, respectively (Table 4). The interspecific distances between these new species and other species range between 19% and 27%, which clearly supports the validity of the species (Table 3).

**Table 3.** Genetic distances (COI) between sequenced species, using the Kimura 2-parameter.

	Species	1	2	3	4	5	6	7	8	9	10	11
**1**	**B. (Baetiella) bibranchia sp. nov.**	0.02										
**2**	B. (Gratia) narumonae	0.21	0.01									
**3**	B. (Gratia) sororculaenadinae	0.22	0.23	-								
**4**	**B. (Baetiella) lannaensis sp. nov.**	0.21	0.25	0.23	0.00							
**5**	**B. (Baetiella) baei sp. nov.**	0.25	0.27	0.26	0.27	0.01						
**6**	B. (Baetiella) bispinosa	0.22	0.24	0.19	0.21	0.25	0.01					
**7**	*B.japonica*	0.22	0.25	0.22	0.25	0.25	0.21	0.02				
**8**	*B.tuberculata*	0.23	0.26	0.24	0.21	0.27	0.24	0.24	0.04			
**9**	*Baetiella* sp. 1	0.24	0.24	0.20	0.24	0.26	0.17	0.20	0.26	0.01		
**10**	*Baetiella* sp. 2	0.21	0.24	0.21	0.23	0.25	0.19	0.22	0.25	0.19	0.00	
**11**	*Cloeondipterum* (Outgroup)	0.19	0.22	0.20	0.24	0.27	0.20	0.23	0.22	0.21	0.22	-

**Table 4.** Comparison of larval morphological characters of three new species of *Baetiella* from Thailand with the closely related species.

Characters/ Species	B. (Baetiella) baei sp. nov.	B. (Baetiella) marginata	B. (Baetiella) muchei	B. (Baetiella) lannaensis sp. nov.	B. (Baetiella) susobskyi	B. (Baetiella) bibranchia sp. nov.	B. (Baetiella) bispinosa	B. (Baetiella) subansiri
Submarginal setae of labrum	1 long medial seta and 1 row of 10 long, robust, simple setae	1 long medial seta and 1 row of 11 robust, simple setae	1 long medial seta and 1 row of 12–22 robust, simple setae	1 long medial seta and 1 row of ≥ 7 robust, simple setae	1 long medial seta and 1 row of < 7 robust, simple setae	1 medial long seta and 1 row of 9 robust, simple setae	1 long medial seta and 1 row of robust, simple setae	1 long medial seta and 1 row of 6–8 robust, simple setae
Edge between mola and prostheca of right and left mandibles	with a row of small spines	smooth without spine	smooth without spine	smooth without spine	smooth without spine	smooth without spine	smooth without spine	smooth without spine
Labial palp	terminal segment rounded, asymmetrical, almost fused with the 2^nd^ segment with small tip at apex; 2^nd^ segment with very small inner apical lobe	terminal segment conical with stout setae and a distinctive tip at apex; 2^nd^ segment with very small inner apical lobe	conical with the apical tip at the apex, 2^nd^ segment with very small inner apical lobe	terminal segment of labial palp conical and symmetrical shaped with apical tip, the 2^nd^ segment of labial palp with small inner apical lobe	terminal segment conical with a distinctive tip at apex; 2^nd^ segment with inner apical lobe	terminal segment conical, rounded and asymmetrical with small tip at apex; 2^nd^ segment without inner apical lobe	terminal segment conical, with a small tip at apex; 2^nd^ segment without inner apical lobe	terminal segment conical with a distinctive tip at apex; the 2^nd^ segment without inner-apical lobe
Thorax	pronotum and mesonotum with small, reduced tubercles	pronotum and mesonotum without tubercles	pronotum and mesonotum without tubercles	pronotum and mesonotum with small, reduced tubercles; metanotum with a single, posteromedian protuberance; surface with dense, numerous rounded scales	pronotum and mesonotum without tubercles	pronotum and mesonotum with small, reduced tubercles	pronotum and mesonotum with distinct tubercles	pronotum and mesonotum with 12 distinct tubercles
Setae of dorsal margin of femur	dense irregular row of long, fine, simple setae; with a pair of long, stout, simple, subapical setae distally	a row of short and simple setae	dense irregular row of long, fine, simple setae	a regular row of long, rounded, simple, ciliated setae, ~1/3 to 1/2 of femur width	a row of dense, long and simple setae on dorsal margin, ~1/2 of femur width	a row of long, rounded, simple setae, ~1/3 of femur width, decreasing at distal part	irregular row of long, dense, fine, simple setae	a row of 17 or 18 long, simple setae, ~1/3 to 1/2 of femur width
Setae of dorsal margin of tibia	irregular row of long, fine, simple setae dorsally	irregular row of dense, fine, simple setae	irregular row of dense, fine, simple setae	irregular row of dense, fine, simple setae	irregular row of dense, fine, simple setae	irregular row of dense, fine, simple setae	irregular row of dense, fine, simple setae	irregular row of dense, fine, simple setae
Coxal gills	absent	absent	absent	absent	absent	Present on forelegs and midlegs	Present on all legs	Present on all legs
Posteromedian protuberances	tergites I–III with small, reduced, single protuberances	absent	absent	tergites I–VIII with single protuberances (decreasing in size towards terminal segment)	tergites I–VIII with single protuberances	tergites I–V with a single, posteromedian protuberance; tergites VI–IX with a pair of posteromedian protuberances	tergites I and II with a single, posteromedian protuberance; tergites III–IX with a pair of posteromedian protuberances	tergites I and II with a single, posteromedian protuberance; tergites III–IX with a pair of a pair of much longer protuberances
Distal margin of tergites	all tergites with multi-dentate, blunt denticles, surface with scattered, fine setae	all tergites with blunt denticles	all tergites with blunt denticles	tergite I smooth without denticles, tergites II–X with multi-dentate and blunt spines; surface with numerous rounded scales	tergites VI–IX with blunt denticles; surface without rounded scale-like setae	tergite I smooth without denticles, tergites II–X with multi-dentate and blunt spines	all tergites with blunt denticles	tergite I–V smooth without denticles, tergites VI–X with blunt denticles
Distal margin of sternites	sternites I–VII smooth without denticles, sternites VIII–X with a row of long, spatulate, blunt denticles	all sternites smooth, without denticles	all sternites smooth, without denticles	sternites I–VII smooth without denticles; sternites VIII–X with multi-dentate, blunt denticles	all sternites smooth, without denticles	all sternites smooth, without denticles	all sternites smooth, without denticles	all sternites smooth, without denticles
Gills	7 pairs of gills; margin with scattered short, small spines; gills II–VII with numerous, fine setae and several micropores on surface	7 pairs of gills; surface with scattered numerous micropores, margin smooth with fine simple setae	7 pairs of gills; surface scattered with numerous micropores, margin smooth with fine simple setae	7 pairs of gills; gill surface with scattered, fine setae and several rounded scales and micropores, gill margin with scattered fine simple setae	7 pairs of gills; surface scattered with numerous micropores, margin smooth with fine simple setae	7 pairs of gills; gill surface with scattered, fine setae and numerous micropores, margin with scattered hair-like setae	7 pairs of gills; margin smooth with fine simple setae	6 pairs of gills; gills II–VI elongate, gill VII reduced; surface with numerous scattered micropores, margin smooth with fine simple setae
Inner margin of paraproct	smooth with 3–5 oval scale-like setae along margin	10–12 stout setae along the inner margin	smooth without denticles	12–14 spines along the inner margin	10–12 spines along the inner margin	several spines along the inner margin	several spines along the inner margin	15–16 scale-like setae along the inner margin
Paracercus	reduced to one segment	reduced to 15 segments	reduced to one segment	reduced to one segment	reduced to 1–3 segments	reduced to one segment	reduced to one segment	reduced to one segment
Distribution	Thailand	India, Nepal, China	Tajikistan	Thailand	India, Nepal	Thailand	China, India, Thailand	India
Reference	This study	Shi and Tong 2015; Vasanth et al. 2020	Braasch, 1978 Sivaruban et al. 2024	This study	Braasch, 1983; Vasanth et al. 2020	This study	Shi and Tong 2015; Phlai-ngam and Tungpairojwong 2018; Vasanth et al. 2020	Vasanth et al. 2020

**Figure 25.**
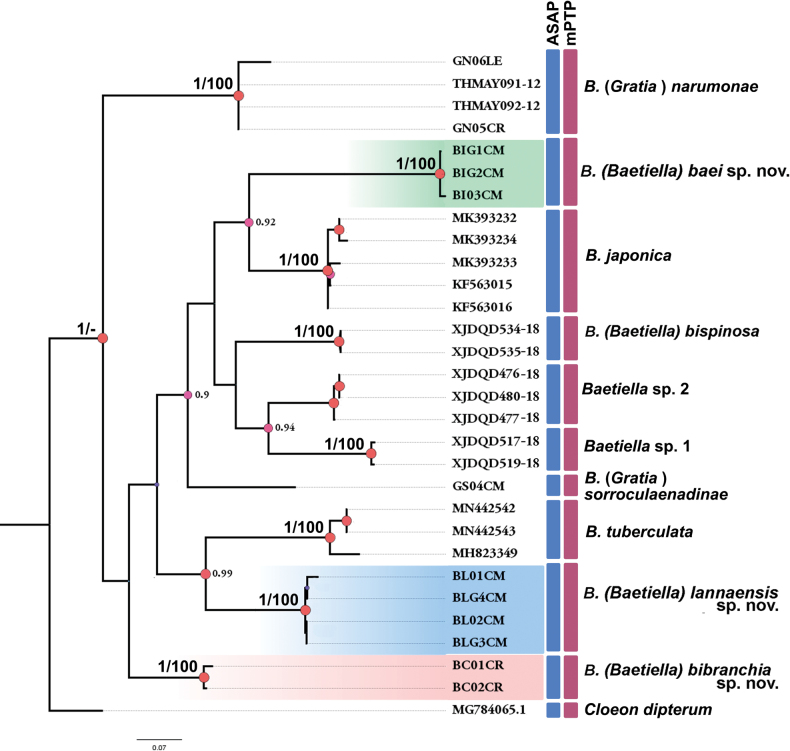
Phylogenetic reconstruction of *Baetiella* species based on Bayesian inference (BI) and Maximum likelihood (ML) analyses of sequences of the mitochondrial COI gene. The highlighted clades represent the newly described *Baetiella* species from Thailand with high bootstrap and Bayesian posterior probabilities (BS/PP) supporting. Pink circles on branches indicate PP > 0.9. Colored vertical boxes indicate species-delimitation hypothesis (i.e., MOTUs) according to the ASAP and mPTP methods.

The utilization of ML and BI analyses in the phylogenetic reconstruction tree of the COI gene reveals that the nine sequences obtained from the newly discovered species can be grouped into three distinct clades. These clades have a significant degree of distinction from other clades, as indicated by their exceptionally high values of bootstrap support (BS = 100%) and posterior probability (PP = 1). In addition, the results indicate that the three *Baetiella* species, B. (Baetiella) bispinosa, B. (Gratia) narumonae and B. (Gratia) sororculaenadinae, are strongly supported as independent clades by high bootstrap values (Fig. 25).

#### ﻿Species delimitation

Species delineation based on molecular evidence has been investigated as an additional method to help define species boundaries (Zhang et al. 2013) and to support phylogenetic results. A set of 29 COI sequences, encompassing *Baetiella* and *Gratia* species, were compiled for the purpose of molecular species delimitation in order to determine species boundaries. The results of the ASAP species delimitation approach, combining the Jukes-Cantor (JC69) model, the Kimura 2-parameter (K2P) model, and simple distances (p-distances), provided a total of 10 Molecular Operational Taxonomic Units (MOTUs). The second approach, mPTP analysis, also provides a total of 10 MOTUs, as depicted in Fig. 25. The ten MOTUs obtained by ASAP and mPTP approaches are the same; both methods recovered B. (Baetiella) baei sp. nov., B. (Baetiella) lannaensis sp. nov., and B. (Baetiella) bibranchia sp. nov. as independent species.

## ﻿Discussion

### ﻿Morphological and molecular studies

Previous studies reported three species of *Baetiella* in Thailand; B. (Baetiella) bispinosa, B. (Gratia) sororculaenadinae, and B. (Gratia) narumonae (Thomas, 1992) (Thomas 1992; Boonsoong et al. 2004; Phlai-ngam and Tungpairojwong 2018). These species were also found during this survey, in addition to the discovery of three new species.

Baetiella (Baetiella) baei sp. nov. presents similarities to *B.marginata* and *B.muchei* in terms of the presence of distinct abdominal protuberances. This newly discovered species can be identified based on several distinct characteristics. These include a small and reduced posteromedian protuberance on the distal margin of tergites I–III, which is not present in other segments. Besides that, the distal margin of sternites VIII–X presents a row of long, spatulate, blunt denticles, and the inner margin of the paraproct is smooth, with 3–5 oval scale-like setae along the margin. Moreover, *B.muchei* shows intraspecific variability larval characters, such as submarginal arc of setae in the labrum which differs from 12 to 22 while B. (Baetiella) baei sp. nov. has no more than 12 submarginal setae on labrum (Sivaruban et al. 2024).

Baetiella (Baetiella) lannaensis sp. nov. shares characters with *B.ausobskyi*, a species found in Nepal (the Himalayan region) and India (Braasch 1983; Shi and Tong 2015b; Vasanth et al. 2020). This new species from Thailand possesses remarkable characteristics, including the presence of ciliated setae on the dorsal margin of the femur as well as different distal margins of the tergites and sternites. The body and gill surfaces are densely covered with rounded scale-like setae. Furthermore, the labrum possesses at least seven subapical setae, while *B.ausobskyi* has less than seven.

Baetiella (Baetiella) bibranchia sp. nov. is easily distinguished from other *Baetiella* species by the presence of a pair of posteromedian protuberances on the distal margin of tergites VI–IX (the distance between posteromedian protuberances gradually increases backwards) and the presence of coxal gills at the base of forelegs and midlegs.

The molecular analysis reveals genetic distances between the species ranging from 17% to 27% (Table 3) and supports the validity of these three newly discovered species. The intraspecific distances for each of the new species are low, ranging from 0% to 2% which is much lower than the generally accepted lower interspecific distance of 4% (Hebert et al. 2003; Ball et al. 2005; Zhou et al. 2010).

This study is the starting point for integrating morphological and molecular data for all species of *Gratia* and *Baetiella* to clarify their systematic. The findings of our study indicate that the morphological structure of Thai *Baetiella* larvae can be assigned to two groups: Baetiella (Baetiella) and Baetiella (Gratia).

Our study reveals imprecisions in part of the previous studies concerning important characters used for the generic delimitation of *Baetiella* and *Gratia*. Some characters are similar between *Baetiella* and *Gratia*, such as the setation of setae on the femur and tibia, labial palp, and posteromedian protuberances. We demonstrate that the shape of submarginal setae on the labrum is a distinct characteristic that can be used for separation of these two baetid groups. However, this single diagnostic character is not sufficient to consider *Baetiella* and *Gratia* as distinct genera; Kluge (2022b) also moved *Gratianarumonae* to *Baetiella* based on the character of the imaginal stage. Even so, the molecular data remain inconclusive. The mitochondrial gene used in our study is valuable for species delimitation but is too variable for deeper nodes. We recommend that researchers apply in the future a combination of molecular and morphological data, encompassing a broader set of species with an increased number of specimens. It will facilitate the gathering of significant molecular data to reconstruct robust phylogeny. Especially, the addition of nuclear genes could provide an effective reconstruction tree for systematic clarification. Even though only the COI gene is usually not enough for phylogenetic analysis. This approach will help to resolve and clarify the taxonomic problems associated with this lineage of baetid mayflies in the future.

This study expands knowledge of *Baetiella* in Thailand, revealing a substantial presence with diverse species mainly in the north of the country. The occurrence of six species, including three new to science, challenges previous expectations of *Baetiella* distribution and diversity and suggest a need for comprehensive diversity surveys in Thailand and more generally in the Southeast Asian region. *Baetiella* species were mainly found in waterfalls and stream with medium or fast current generally close to headwaters. Larvae were predominantly found in cobbles, boulders, and bedrock in cold, pristine water. The ecological data gathered could contribute to freshwater quality monitoring, challenging the too often accepted paradigm that Baetidae in general are not sensitive to water quality and have a broad ecological valence.

### ﻿The geographic distribution and global status of *Baetiella*

**Figure 26.**
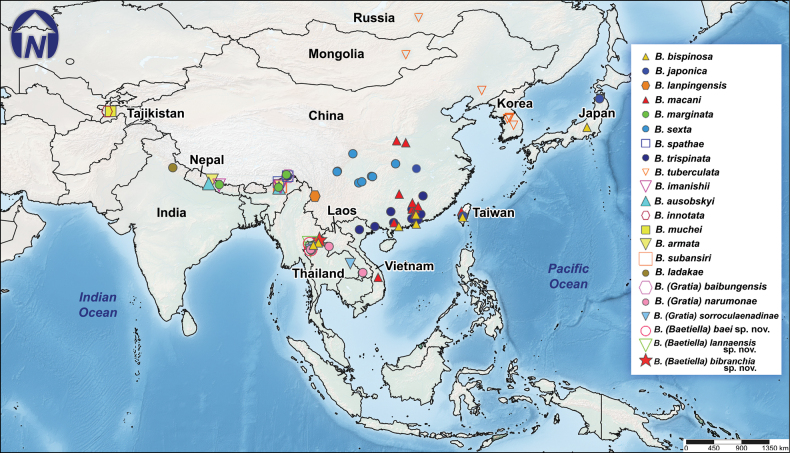
Distribution map of *Baetiella* species in the world.

The genus *Baetiella* has been recorded in the Eastern Palearctic and Oriental regions (Fig. 26). Most species are dispersed within the Palearctic realm, encompassing several countries such as China, Japan, Korea, Russia, Mongolia, Tajikistan, Vietnam, Nepal, and India (Tong and Dudgeon 2000; Shi and Tong 2015a; Vasanth et al. 2020).

Currently, *Baetiella* comprises 22 valid species, including the newly discovered species and the species previously assigned to *Gratia*. Despite the increasing number of new species found and the new reports, the distribution of this genus remains limited to these two zoogeographic zones.

The highest diversity has been reported in China, primarily on the mainland. These species include *B.sexta* Shi & Tong, 2015, *B.macani*, *B.marginata*, *B.trispinata*, *B.bispinosa*, *B.imanishii*, and *B.lanpingensis*. A few species are reported from Hong Kong (*B.bispinosa* and *B.trispinata*). Others are distributed in high mountains; *B.spathae* and *B.lanpingensis* are found in Tibet and Yunnan, respectively (Tong and Dudgeon 2000; Shi and Tong 2015a). The size of the country and the various ecological conditions contribute to the species richness of *Baetiella* in China.

Other *Baetiella* species, including *B.japonica*, *B.bispinosa*, and *B.macani*, are mentioned from islands such as Japan and Taiwan. *Baetiellatuberculata* exhibits a broad distribution across several Palearctic countries, including Russia, Mongolia, China, and Japan (Uéno 1969; Müller-Liebenau 1985; Shi and Tong 2015a).

Several species (e.g., *B.armata*, *B.aubobskyi*, *B.imanishii*, *B.marginata*, *B.spathae*, and *B.subansiri*) are found in the southern limit of the East Palearctic region as they are only reported from north India and Nepal. *Baetiellamuchei* and *B.innotata* Braasch, 1978 were collected in Tajikistan, with no report of these species in any other regions (Braasch 1978, 1983; Vasanth et al. 2020). The currently accessible data indicate that the distribution of *Baetiella* species is limited to the eastern part of the Palearctic region.

Most species occur mainly in the northern part of the Oriental realm, arguing for possible dispersal routes ranging from northern Vietnam to northeastern Thailand. This point needs to be assessed against the lack of knowledge from some parts of the Oriental realm and especially from equatorial and lower equatorial countries.

Our study demonstrates that the distribution and diversity of *Baetiella* was highly underestimated in Thailand. We have not only greatly extended the distribution of Palearctic species southwards, but also discovered species that are potentially endemic to northern Thailand.

Based on our in-depth study of the fauna of Thailand and initial data from Laos and Vietnam, we can reasonably expect to find similar diversity in neighboring countries such as Cambodia and Myanmar, where knowledge of the fauna is still very patchy. Given the current state of our knowledge, it is difficult to predict whether the genus *Baetiella* will reach its southern limit in Thailand or whether we can expect to discover populations further south (e.g., Malaysia, Java, Sumatra or Borneo).

## Supplementary Material

XML Treatment for Baetiella (Baetiella) baei

XML Treatment for Baetiella (Baetiella) lannaensis

XML Treatment for Baetiella (Baetiella) bibranchia

XML Treatment for Baetiella (Baetiella) bispinosa

XML Treatment for Baetiella (Gratia) narumonae

XML Treatment for Baetiella (Gratia) sororculaenadinae

## ﻿Additional information

### Author ORCIDs

Sirikamon Phlai-ngam https://orcid.org/0000-0002-3638-6123

Boonsatien Boonsoong https://orcid.org/0000-0002-8166-0021

Jean-Luc Gattolliat https://orcid.org/0000-0001-5873-5083

Nisarat Tungpairojwong https://orcid.org/0000-0001-8135-4246

### Data availability

All of the data that support the findings of this study are available in the main text.
